# DFT-Guided Design and Fabrication of Carbon-Nitride-Based Materials for Energy Storage Devices: A Review

**DOI:** 10.1007/s40820-020-00522-1

**Published:** 2020-10-29

**Authors:** David Adekoya, Shangshu Qian, Xingxing Gu, William Wen, Dongsheng Li, Jianmin Ma, Shanqing Zhang

**Affiliations:** 1 Centre for Clean Environment and Energy, School of Environment and Science, Griffith University, Gold Coast Campus, Gold Coast, QLD 4222 Australia; 2 College of Materials and Chemical Engineering, Key Laboratory of Inorganic Nonmetallic Crystalline and Energy Conversion Materials, China Three Gorges University, Yichang, 443002 People’s Republic of China; 3 Key Laboratory of Materials Processing and Mold (Zhengzhou University), Ministry of Education, Zhengzhou, People’s Republic of China

**Keywords:** Carbon nitrides, Metal-ion batteries, Density functional theory, g-C_3_N_4_, Anode

## Abstract

Carbon nitrides (including CN, C_2_N, C_3_N, C_3_N_4_, C_4_N, and C_5_N) are a unique family of nitrogen-rich carbon materials with multiple beneficial properties in crystalline structures, morphologies, and electronic configurations. In this review, we provide a comprehensive review on these materials properties, theoretical advantages, the synthesis and modification strategies of different carbon nitride-based materials (CNBMs) and their application in existing and emerging rechargeable battery systems, such as lithium-ion batteries, sodium and potassium-ion batteries, lithium sulfur batteries, lithium oxygen batteries, lithium metal batteries, zinc-ion batteries, and solid-state batteries. The central theme of this review is to apply the theoretical and computational design to guide the experimental synthesis of CNBMs for energy storage, i.e., facilitate the application of first-principle studies and density functional theory for electrode material design, synthesis, and characterization of different CNBMs for the aforementioned rechargeable batteries. At last, we conclude with the challenges, and prospects of CNBMs, and propose future perspectives and strategies for further advancement of CNBMs for rechargeable batteries.

## Highlights

- Comprehensive summary of crystalline structures and morphologies of carbon nitride-based materials (CNBMs).
- Density functional theory computation for the design of functional CNBMs for rechargeable battery applications.
- The experimental synthesis strategies of CNBMs for rechargeable battery application.

## Graphical abstract

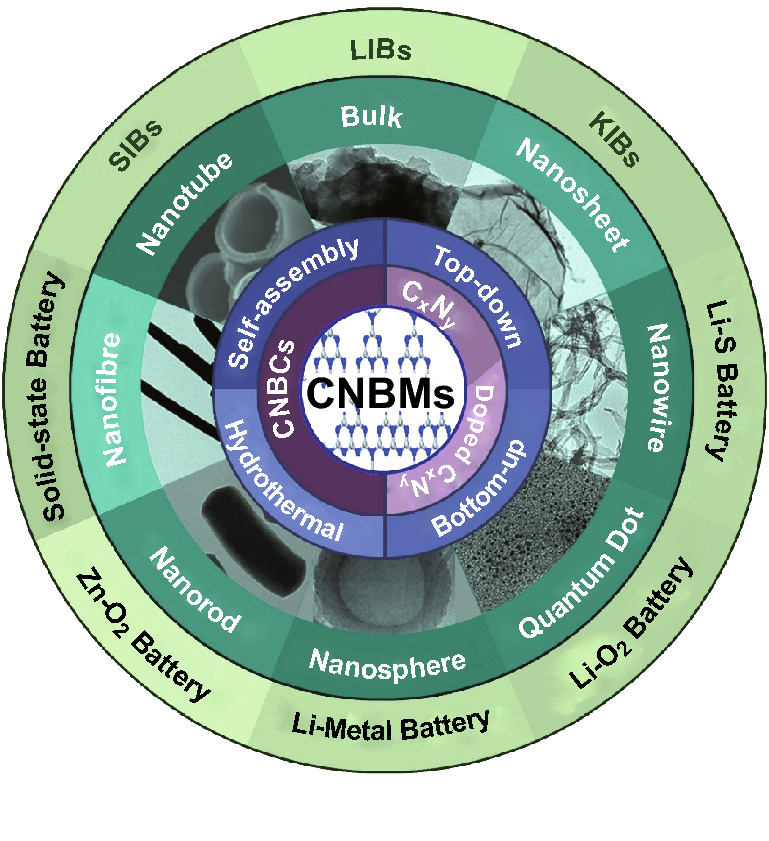

## Introduction

Rechargeable metal ion batteries (MIBs) are one of the most reliable portable energy storage devices today because of their high power density, exceptional energy capacity, high cycling stability, and low self-discharge [1, 2]. Lithium-ion batteries (LIBs) remain the most developed and commercially viable alternative among all rechargeable batteries, and graphite is widely accepted as a preferred negative electrode (anode) material for LIBs [3]. Graphite is affordable, and it does not suffer significant volumetric changes (compared to other metal-based electrodes); it operates at a very low voltage close to that of lithium metal (~ 0.1 V) and exhibits moderately stable cycle life with a theoretical capacity of 372 mAh g (LiC_6_). Despite these positive characteristics, the performance of graphite is still very limited, and one of the effective ways to modulate its properties and electrochemical performance is through nitrogen doping [4]. Precisely, the work of Liu et al. showed that the N-doping of graphene results in improved conductivity and charge transfer, which boosts its performance and cyclability [5].

Carbon nitrides are a family of nitrogen-rich graphite analogues which contain a high nitrogen content and porous defect sites for effective charge transfer in energy storage devices [6, 7]. However, carbon nitrides are limited by poor electrical conductivity, chemical inertness, and ineffective intercalation/deintercalation process [8]. Due to these issues, several research studies have focused on the design of unique carbon-nitride-based materials (CNBMs), including pure carbon nitrides, doped carbon nitrides (DCNs) as well as carbon-nitride-based composites (CNBCs) [9]. Most of these reports have focused on g-C_3_N_4_ because it is easy to synthesize, low cost, environmentally safe, and it has a theoretical capacity of 524 mAh g^−1^ (Li_2_C_3_N_4_). In contrast, though other carbon nitride structures (such as CN, C_2_N, C_3_N, C_4_N, and C_5_N) which have been studied through density functional theory (DFT) calculations exhibit exceptional structural and electronic properties which demonstrates their capabilities as promising MIB electrode materials, they have attracted less attention.

**Fig. 1:**
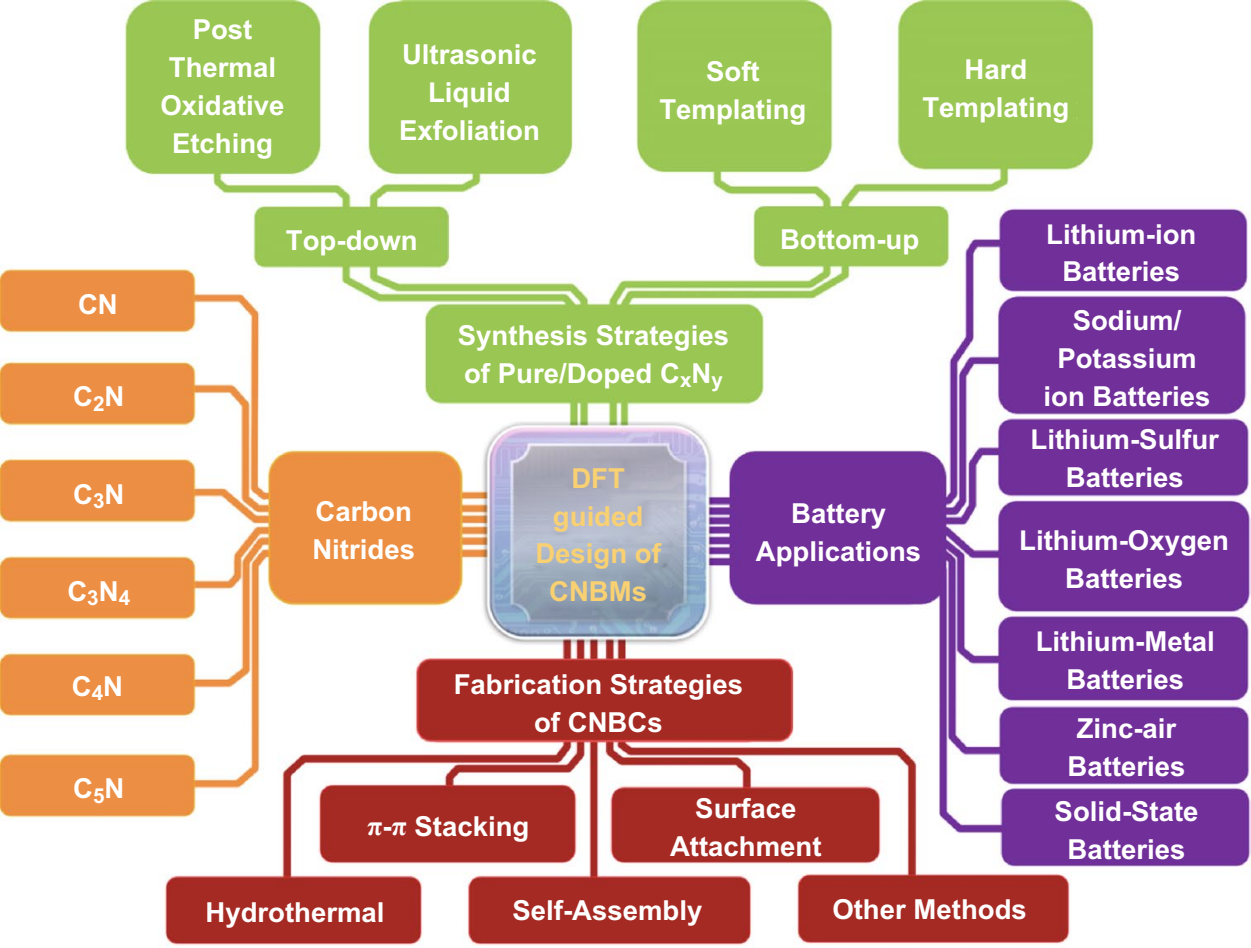
Overview of the main topics of this work, including DFT-guided design (symbolized by the “CPU” at the center), the molecular configuration of carbon nitrides (yellow block), the synthesis strategies of pure/doped carbon nitrides (green block), the fabrication strategies of CNBCs (red block) and the battery applications of CNBMs (purple block). (Color figure online)

On the other hand, DFT has been adopted more and more in studying CNBMs and their electronic properties [10]. In fact, DFT could provide effective guidance for the synthesis of electrode materials and/or interpretation of the structure–performance relationship in energy storage devices, including LIBs, sodium- and potassium-ion batteries (SIBs and PIBs), lithium-sulfur batteries (Li–S), lithium-oxygen (Li-O_2_) batteries, lithium metal batteries (LMBs), zinc air batteries (ZABs), and solid-state batteries (SSBs). This review aims to comprehensively discuss the relationships between the structural and electronic properties and the MIB performance of pure carbon nitrides, doped carbon nitrides, and CNBCs, summaries the theoretical computation for the design of functional CNBMs for different rechargeable MIB applications, and generalizes the synthesis strategies of pure carbon nitrides and CNBCs for rechargeable metal ion battery application (Fig. 1). At the end of this work, we also offer a perspective on the existing challenges of carbon nitrides for energy storage devices and relevant resolving strategies.

## CNBMs

**Fig. 2:**
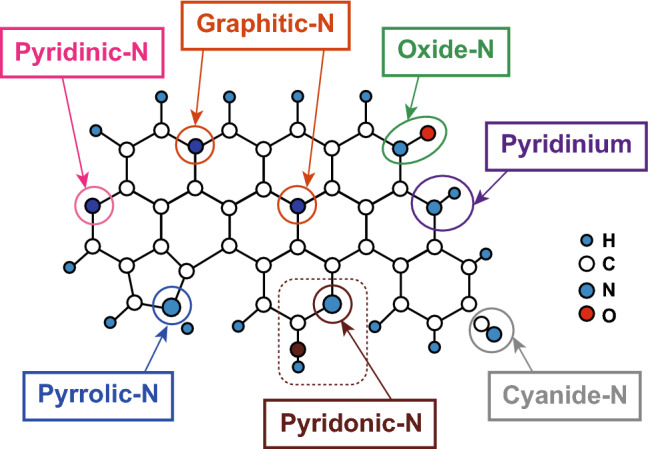
Common forms of nitrogen species in nitrogen-doped carbon materials. Reproduced with permission from Ref. [12]. Copyright permissions from Wiley–VCH. (Color figure online)

To study the carbon nitrides, we must begin by exploring their crystalline structure, structural and electronic properties, and functionality. Carbon nitrides are a family of nitrogen-rich carbon materials, and they have different crystalline structures and molecular configurations. Generally, there are seven types of nitrogen species (Fig. 2), with at least two most common species of nitrogen (the graphitic-N and pyridinic-N). The nature of the nitrogen and its percentage concentration has been proven to impact the electronic configuration and characteristic of the carbon nitride for different applications. The classification of carbon nitrides is often based on the carbon to nitrogen content (i.e., the C/N ratio) which is often associated with the degree of surface defects in their structure [11]. The carbon nitrides discussed in this review are categorized based on the dominant N species in their crystalline structure.

### Pure Carbon Nitrides

**Fig. 3:**
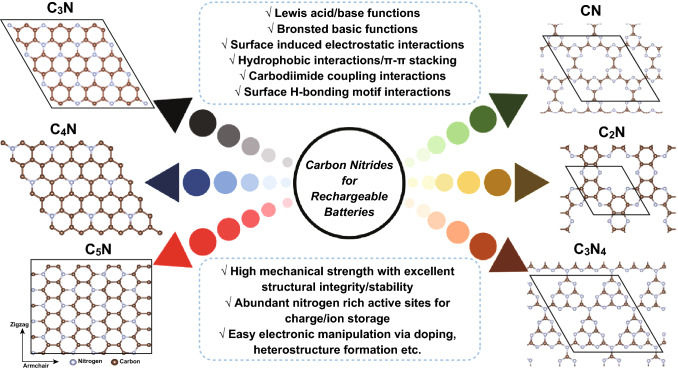
Geometric structures of the pyridinic and graphitic-N-based carbon nitrides reported for rechargeable batteries and their surface functionalities. The brown spheres represent carbon atoms, and the light blue one represents nitrogen atoms in the 2D carbon nitrides structures. (Color figure online)

Based on their generic family molecular formula of C_x_N_y_, the carbon nitride family includes CN (C_2_N_2_, C_3_N_3_, C_4_N_4_), C_2_N, C_3_N, C_3_N_2_, C_3_N_4_, C_3_N_5_, C_3_N_6_, C_3_N_7_, C_4_N, C_4_N_3_, C_5_N, C_6_N_6_, C_6_N_8_, C_9_N_4_, C_9_N_7_, C_10_N_3_, C_10_N_9_, and C_14_N_12_ [11, 13]. All these carbon nitride materials possess some similarity with graphene in that they are all two-dimensional (2D) materials with *sp*^2^/*sp*^3^ hybridized conjugated C atoms [14], but they all exhibit different structural frameworks and C/N ratio. In this section of the review, we summarize the crystalline structure, surface functionalities, and electronic properties of different carbon nitrides, including pyridinic nitrogen-based carbon nitrides (CN, C_2_N, and C_3_N_4_) and graphitic nitrogen-based carbon nitrides (C_3_N, C_4_N, and C_5_N) (Fig. 3).

#### Pyridinic Nitrogen-Based Carbon Nitrides (CN, C_2_N, C_3_N_4_)

Pyridinic-N is one of the edge site nitrogen species in an N-doped carbon material or carbon nitride. It is bonded to two carbon atoms and supplies one p electron to the *π*–*π* conjugated system of carbon [12]. Due to the co-ordination of pyridinic-N to 2 carbon atoms, it donates 4 electrons to the *sp*^*2*^ of each carbon atom along with a lone pair. Because of its superior electronegativity, it is able to pull π-electrons from the conjugated system of carbon, thereby making it negatively charged N-atom. When the π-conjugated system of carbon is broken (the aromaticity), a non-bonding *p*_*z*_ orbital is formed, and this orbital can also be observed around defected carbon materials. Carbon materials that contain pyridinic-N can function as Lewis base because the pyridinic-N exhibits a negative charge on surrounding carbon atom, which makes them positively charged with a lone pair of electrons as a result of the non-bonding *p*_*z*_ orbital [12]. The pyridinic-N-based carbon nitrides in this category include CN, C_2_N, and C_3_N_4_.

##### CN

**Fig. 4:**
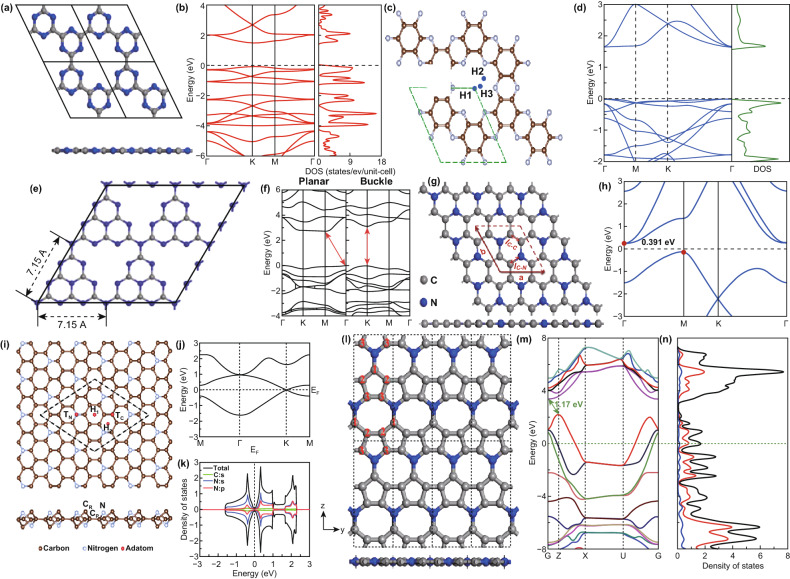
**a** Top view and side view of the supercell (2 × 2) g-C_3_N_3_. **b** Band structure and total density of state for 1 × 1 g-C_3_N_3_. Reproduced with permission from Ref. [15]. **c** Relaxed structure of 2  ×  2 C_2_N monolayer, H1, H2, and H3 are possible binding sites for transition metal atom doping on the C_2_N. **d** Band structure and density of states of C_2_N monolayer. Reproduced with permission from Ref. [28]. **e** Schematic structure of monolayer g-C_3_N_4_. Reproduced with permission from Ref. [18]. **f** Calculated band structures of monolayer g-C_3_N_4_ with planar or buckled topology. Reproduced with permission from Ref. [20]. **g** Optimized structure and **h** band structure of C_3_N monolayer. The unit cell is shown by the red dashed line. Reproduced with permission from Ref. [29]. **i** Top and side view of the atomic structure of monolayer C_4_N. The black dashed lines show the 3 × 3 × 1 supercell of monolayer C_4_N. Four adsorption sites were considered (H_1_, H_2_, T_C_, T_N_). **j** Electronic band structures and k PDOS of the unit cell of a pristine C_4_N monolayer. Reproduced with permission from Ref. [30]. l Top (upper) and side (lower) view of the atomic structure of C_5_N monolayer. The gray and blue balls represent C atoms and N atoms, respectively. m Band structure and n density of states (DOS) of C_5_N monolayer obtained from HSE06 calculations. The black, red, and blue lines denote the total DOS of C_5_N, the partial DOS of C atoms, and the partial DOS of N atoms, respectively. Reproduced with permission from Ref. [27]. Copyright permissions from Elsevier, Royal Society of Chemistry and Wiley–VCH. (Color figure online)

CN exists in different compositions such as C_2_N_2_, C_3_N_3_, and C_4_N_4_, and the structure of these carbon nitride materials possesses round and uniform pores, six pyridinic nitrogens in each unit cell and a bandgap of ~ 1.5–1.6 eV (Fig. 4a, b) [15]. Most of the carbon nitrides contain both graphitic and pyridinic nitrogen except for CN. Due to its rich pyridinic nitrogen structure and absence of graphitic nitrogen, CN exhibits high structural stability, better conductivity and does not experience loss of crystallinity when it interacts with alkali metal ions like Li^+^.

##### C_2_N

The structure of C_2_N exhibits *sp*^2^ hybridization, and it is filled with uniformly sized holes and one large hole at the center, which is due to the six-member nitrogen-containing ring (Fig. 4c). Like the structure of graphene, it possesses several benzene rings that are bridged by pyrazine rings, which have two nitrogen atoms opposite to each other [16]. Also, unlike graphene, which has a fully conjugated π–electron structure, the presence of nitrogen in the structure of C_2_N makes the *π*–electronic structure of its benzene rings isolated. Therefore, it has a flat band and exists as a semiconductor with a bandgap of 1.96 eV (Fig. 4d). Due to its wider structure and the abundance of several benzene and pyrazine rings, it is expected that it will possess several active sites for alkali metal ion storage. Moreover, the defect sites and higher nitrogen population of C_2_N suggest that it will exhibit superior conductivity to boron nitride (BN) [17].

##### C_3_N_4_

C_3_N_4_ carbon nitride is the most reported member of the carbon nitride family. It is composed of consistently repeated tri-s-triazine units having planar *sp*^*2*^-hybridized conjugation structures held together by van de Waals forces. It often occurs as bulk C_3_N_4_, which has a relatively low indirect bandgap of ~ 2.7 eV and an interplanar distance of 0.324 nm and can be functionalized to obtain other architectures such as the monolayer 2D sheet (Fig. 4e) [18]. This indirect bandgap of C_3_N_4_ highly has spurred massive interest in its application for several applications such as photocatalysis, oxygen evolution, reduction reactions, sensing devices, etc. However, the semiconductor property of bulk C_3_N_4_ limits the electronic conductivity for application in electronic devices [19]. Under buckling, the indirect bandgap of bulk C_3_N_4_ can be tuned into a direct bandgap material with superior electronic conductivity (Fig. 4f) [20]. This ability to easily modify the electronic and structural properties of C_3_N_4_ makes it one of the most preferred and widely applied carbon nitride structures [21].

#### Graphitic Nitrogen-Based Carbon Nitrides (C_3_N, C_4_N, and C_5_N)

Graphitic nitrogen is one of the most widely known forms of nitrogen. It has a nitrogen bonded to three carbon atoms, and it is the nitrogen-doped into the basal graphitic carbon plane of the material. Due to its superior negativity in comparison with carbon, it is possible for graphitic nitrogen to induce a positive charge on adjacent carbon, thereby making it readily active site for attraction to negatively charged species [22]. This makes it a commonly desired form of nitrogen doping, just like pyridinic nitrogen. The graphitic-N-based carbon nitrides, which we shall discuss in this review, include C_3_N, C_4_N, and C_5_N.

##### C_3_N

The structure of C_3_N contains well-defined homogenous rings with no large holes. It is a 2D honeycomb lattice configuration that displays a D_6h_-symmetry that facilitates high structural stability and superior thermal conductivity (Fig. 4g). It is a semiconductor which displays a unique molecular orbital with a low unoccupied molecular orbital gap (~ 2.7 eV) and an indirect bandgap of 0.39 eV (Fig. 4h) [23].

##### C_4_N

Compared to the atomic structure of another carbon nitride, the C_4_N structure is filled with *sp*^3^ hybridized species and due to the presence of 4 *p*_*z*_ atomic orbitals in the projected electron density of states (PDOS), it exhibits a Dirac cone shape in its band structure like that of graphene (Fig. 4i, j). It is often called the dumbbell (DB) C_4_N and depending on the positions of the raised C/N atoms in its structure, two structural configurations exist, namely the DB C_4_N-I and the DB C_4_N-II. The former refers to a C_4_N monolayer structure in which the raised C/N atoms are located on the same side, while the latter is a C_4_N monolayer structure where C/N atoms are on opposite sides [24, 25]. These two configurations of C_4_N are semiconductors in nature and display a narrow and zero bandgap (Fig. 4k); however, this property can be tuned easily [24, 26].

##### C_5_N

C_5_N is the most recently reported graphitic-N-based carbon nitrides; like C_4_N, the atomic structure of C_5_N is filled with *sp*^3^ hybridized carbon atoms; however, it contains two cyclic rings of different sizes (Fig. 4l). A 5-membered carbon ring and an eight-membered carbon ring with two nitrogen atoms are opposite to each other, and these rings are side-by-side to each other (see the atomic structure of C_5_N in Fig. 4k). The C_5_N structure has some interesting features such as high chemical, mechanical and thermodynamic stability. Also, analysis of the band structure shows that the Fermi level is located below the valence-band maximum, which suggests it will be metallic and display superior conductivity than other carbon nitrides (Fig. 4m, n) [27].

### Doped Carbon Nitrides

It has been proven that the metal ion storage capacity of an electrode material such as carbon nitride is significantly dependent on the electronic property. Most carbon nitrides are semiconductors, the electronic density of states (DOS) of the majority of the carbon nitrides possess a lone pair orbital of nitrogen at their Fermi level, and this leads to a large effective mass for hole/electron and results in poor electronic conductivity [19]. Therefore, doping is important for carbon nitride structures. Heteroatoms of elements at the top end of the periodic table such as sulfur, nitrogen, boron, and phosphorus, display narrow bandgap and lower high occupied molecular orbital, which signifies superior electronic conductivity. Therefore, replacing some of the carbon atoms in the crystalline structure of carbon nitrides with metallic or non-metallic atoms can boost their electronic conductivity and electron mobility. Doping also creates surface defects on the carbon nitride structure, and this is beneficial for effective charge transport.

**Fig. 5:**
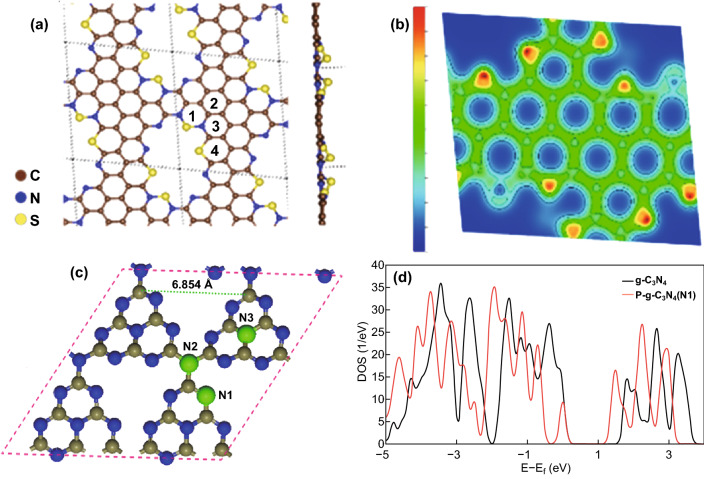
**a** Optimized atomic structure of S-doped mesoporous CN (S-MCN). **b** Charge density profile of S-MCN, blue color—lowest electron density (0%), and red color—highest electron density (100%). Reproduced with permission from Ref. [31]. **c** Optimized structure g-C_3_N_4_ monolayer, showing possible P-doping sites. **d** DOS plots of P-doped g-C_3_N_4_ for P substitution at N1 and N2 sites. Reproduced with permission from Ref. [32]. Copyright permissions from American Chemical Society and Springer Nature. (Color figure online)

Three heteroatoms are commonly used to dope carbon nitrides. These are carbon, nitrogen, sulfur, and phosphorus. C doping in carbon nitrides can facilitate the formation of delocalized *π*-bonds, which can result in *n* − π* electronic transition due to interaction with inherent nitrogen, and this will boost the electronic conductivity and charge transfer. N and S doping have proven to enlarge the interlayer distance of carbon nitrides; this facilitates the adsorption of metal ions and improves the conductivity. Cha et al. reported S-doped mesoporous CN carbon nitride (Fig. 5a shows the optimized structure), and from the electron density profile presented in Fig. 5b, it is obvious that effective charge distribution can be achieved at S-doped sites [31]. Boron (B) and phosphorus (P) doping can decrease the indirect bandgap of semiconductor carbon nitrides and make them metallic. Molaei et al. studied the effect of doping C_3_N_4_ with phosphorus by replacing some of the N atoms with P atoms (Fig. 5c), and the DOS result shown in Fig. 5d confirms a shift of the Fermi level toward the conduction band. This suggests a narrowed band gap and enhanced electronic conductivity compared to pure C_3_N_4_ [32]. In summary, heteroatom doping of carbon nitrides is beneficial for boosting their electrical conductivity, improving charge transfer, enlarging their interlayer distance, and boosting metal ion storage.

### CNBCs

Although doping is an effective way of modulating the electronic conductivity of carbon nitrides, they are still limited by poor reversible capacity due to ineffective metal ion storage mechanisms and inadequate surface area. Most carbon nitrides are not able to achieve the desired performance in battery testing because the adsorption energy may be too strong for effective adsorption and desorption of metal ions or the diffusion barrier may be too high for easy metal ion transport. Also, apart from poor electronic conductivity, pure carbon nitrides are characterized by smaller surface area, which provides inadequate coverage for metal ion adsorption. CNBCs can solve these problems because combining pure carbon nitride with highly conductive materials like reduced graphene oxide (rGO) will produce a material with improved electronic conductivity and larger surface area. Moreover, the formation of CNBCs with high theoretical capacity metal oxides such as Fe_2_O_3_ will provide an anode material with an improved reversible capacity [33]. CNBCs can also be formed by combining pure carbon nitride with metal chalcogenides, perovskites, etc., for different metal-ion batteries. Hence, we summarize that an effective way to resolve the problems of poor reversible capacity and limited surface area is through CNBCs.

## DFT-Guided Studies of CNBMs for Energy Storage Devices

**Fig. 6:**
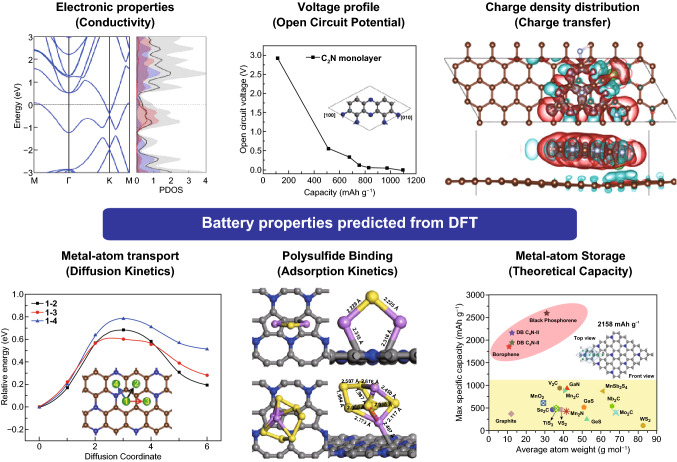
Electrochemical properties of carbon nitrides can be predicted from DFT calculations. Reproduced with permission from [27, 37–40]. Copyright permissions from Elsevier, American Chemical Society, Wiley–VCH, and IOP Publishing. (Color figure online)

After the first discovery/synthesis of tri-*s*-triazine-based carbon nitride by Berzelius in 1834, the prediction and synthesis of beta-C_3_N_4_ in 1993, the discovery of other carbon nitride structures have largely depended on theoretical first-principle simulations. First-principle calculations allow researchers to calculate or predict the structures and properties of material before its synthesis. It can also be used to resolve the discrepancy between experimental measurements and theoretical calculations by considering various possible alkali-metal atom diffusion kinetics, including diffusions in the bulk structure, on the surface, in the defect rich sites, etc. [1, 34]. This prevents random experimental tests or synthesis, thereby facilitating materials discovery. Furthermore, modification strategies for improving the properties of prospective materials can be easily explored through first-principle calculations. DFT is one of the most common computational simulation methods. It is a very powerful tool used to predict different electrochemical parameters of electrode materials. DFT calculations can be used to determine the alkali-metal atom insertion voltage of electrode materials, calculate the migration energy barriers of the alkali-metal atom, and directly visualize the transport pathways and dynamics [35, 36]. In addition, the theoretical capacity of a potential electrode material can be predicted from DFT calculations by investigating the maximum metal atom loading on its structure. The adsorption energy of important molecules on the structure of electrode materials can also be calculated; for example, the adsorption of polysulfides on Li–S cathode materials. Crystal and surface defects are important properties of an electrode material because they impact the alkali-metal atom transport mechanism and pathways, and their effect can also be studied through first-principle calculations. Moreover, information on the intrinsic electronic conductivity and bandgap can be directly inferred from the calculated electronic density of states by observing the Fermi level, conduction, and valence bands. The important role of DFT calculations in CNBMs for energy storage devices includes the DFT-guided synthesis and the DFT-predicted electrochemical properties. In terms of DFT-guided synthesis, DFT studies can determine important synthesis parameters such as the formation energy, exfoliation energy, and cohesive energy of a CNBM. These are criteria that indicate the ease/feasibility of synthesizing a CNBM experimentally. DFT calculations can also provide an atomistic understanding of the interaction between the C/N and the adsorbed metal atom, which will reveal the structural limitations of the CNBMs and guide the synthesis of superior material. DFT studies also play a significant role in predicting fundamental electrochemical properties such as the bandgap, metal atom adsorption energy, open-circuit potential, charge transport kinetics, and theoretical capacity of the CNBMs. These parameters forecast the prospect of CNBMs for energy storage devices and reveal potential limitations that must be considered and improved upon. The electrochemical properties that can be predicted from DFT are summarized in Fig. 6.

### Theoretical Calculations on CNBMs for Energy Storage Devices

Density functional theory calculations are one of the most useful tools for discovery and electrochemical investigation of different carbon nitrides for rechargeable batteries [41]. In this section, we discuss different carbon nitrides that have been studied through DFT.

#### Pure Carbon Nitrides

**Fig. 7:**
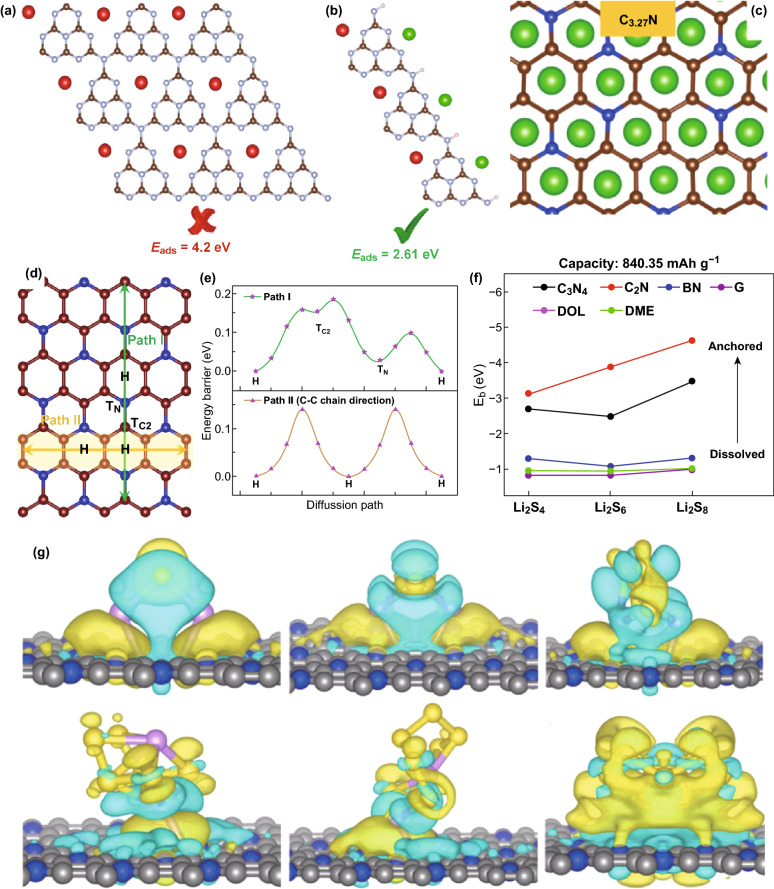
Reversible Li active sites in **a** 2D-C_3_N_4_ sheet, and **b** 1D-C_3_N_4_ fiber. Green means reversible Li atoms and red means non-reversible Li atoms. **c** Top and side view of stable intercalation structures of *n* Li ions into C_3_N. Reproduced with permission from Ref. [40]. **d** Diffusion paths and **e** corresponding energy barriers of Li migration in C_3_N-S2. Reproduced with permission from Ref. [51]. **f** Binding energy of Li_2_S_4_/Li_2_S_6_/Li_2_S_8_ interacting with G, BN, C_2_N, C_3_N_4_ and DOL/DME solvent, respectively. Reproduced with permission from Ref. [17]. **g** Isosurfaces of charge density difference of Li_2_S, Li_2_S_2_, Li_2_S_4_, Li_2_S_6_, Li_2_S_8_, and S_8_ adsorbed on the surface of C_5_N with the isovalue of 0.003 A^−3^. Blue wireframes denote loss of electrons and yellow wireframes denote gain of electrons. Reproduced with permission from Ref. [27]. Copyright permissions from American Chemical Society, Elsevier and Wiley–VCH. (Color figure online)

Through DFT calculations, it was predicted that C_3_N_4_ carbon nitride could deliver a theoretical capacity of up to 524 mAh g^−1^ (Li_2_C_3_N_4_) [42], and this capacity has not been achieved in battery testing. Mao et al. [43], Veith et al. [44], and Hankel et al. [45] showed that this poor performance was due to the high content of graphitic nitrogen in its structure which resulted in ineffective intercalation. We reported the first C_3_N_4_ with reduced graphitic-N and increased pyridinic-N for LIBs, and our DFT calculations showed that unlike bulk/sheet C_3_N_4_ which adsorbs Li atom in the triangular pore at high energy of ~ 4 eV, a 1D-C_3_N_4_ fiber possesses lower Li binding energy (2.61 eV) at its edges. Hence, it can effectively adsorb/desorb Li atoms (Fig. 7a, b) [46]. Hui Pan reported a first-principle study on C_3_N_4_ carbon nitride nanotubes for LIBs and predicted that it could store Li atoms internally and externally due to its porous structure, which might make it better than other dimensions of carbon nitride [47].

Other carbon nitride structures have also been studied for LIBs through DFT. For example, Hankel et al. showed that although g-CN can deliver a high capacity of 454 mAh g^−1^. Its high Li binding energy of > 3 eV leads to ineffective intercalation/deintercalation and limits its prospects for LIBs [48]. The study of Hussain et al. on C_2_N-*h*2D showed that despite the pyridinic nitrogen, which facilitates Li storage, the high initial Li adsorption energy would result in poor battery performance [16]. By using a “pair of particle (metals) model,” Wu et al. [49] carried out a DFT study on C_2_N for LIBs and SIBs to show that C_2_N monolayer can achieve a capacity of 2939 and 2469 mAh g^−1^ for LIBs and SIBs with low diffusion barrier and an OCV of 0.45 V [16]. Liu et al. carried out a DFT study on different C_3_N compositions (C_3_N, C_2.67_N, and C_3.33_N) and showed that the drop in capacity experience by Xu et al. [50] was due to ineffective intercalation of Li. They concluded that C_3.33_N is the preferred C_3_N composition, and it delivered a reversible capacity of 840.35 mAh g^−1^ (Fig. 7c), operated at a low open-circuit potential of 0.12 V, and displayed superior electronic conductivity [40]. Guo et al. proposed other C_3_N allotropes (C_3_N-S1, C_3_N-S2, and C_3_N-S3) with unique electronic properties, and the C_3_N-S2 structure displayed the most feasible Li diffusion pathway with lower diffusion barrier (0.14 eV) for LIBs (Fig. 7d, e) [51]. Yang et al. also reported the first-principle study of two structural configurations of DB C_4_N as LIBs anode for LIBs, and they predicted that DB C_4_N-I and DB C_4_N-II could deliver high theoretical capacities of 1942 mAh g^−1^ (DB C_4_N-I) and 2158 mAh g^−1^ (DB C_4_N-II) [52].

DFT studies of pure carbon nitrides for large-sized metal ion batteries have also been reported. For instance, Weng et al. showed that pure C_3_N_4_ nanosheet displays high adsorption energy for Na-atom, but it shows an exceedingly high diffusion barrier, which makes it fail as a SIBs anode [53]. Moreover, through DFT we showed that thanks to the high pyridinic-N of 1D-C_3_N_4_ fiber it exhibits a high affinity for potassium ions, but it suffers from a rather high K-diffusion barrier which limits effective ion transport [54]. Bhuriyal et al. also reported the first-principle study of C_3_N monolayer as a promising anode material for SIBs and PIBs, and they showed that multilayer adsorption of the metal atoms generated a high capacity of 1072 mAh g^−1^ and low diffusion barrier of 0.03 and 0.07 eV for Na and K-ion [55]. Xu et al. showed that metal ion (Li, Na, and K) adsorption on C_4_N is an effective way to open the zero bandgap and modulate the electronic property because the adsorbed metal ion transfers charges to the surface of DB C_4_N [30].

Thanks to the nitrogen-rich structure, carbon nitrides have also been studied for Li–S and other battery systems. Li et al. reported that due to the accumulation of charges at the N–N bond of the C_4_N_4_ structure, it demonstrates superior lithium polysulfide species (LIPSs) anchorage than commercial electrolyte solvent molecules (DOL and DME) [56]. Zheng and co-workers compared the LIPSs anchoring property of graphene, boron nitride (BN), C_2_N, and C_3_N_4_ with commercial solvents and showed that C_2_N and C_3_N_4_ were the most effective due to their stronger binding energy (Fig. 7f) surface interaction with LiPSs via the Li-N/C-S bonds formed during their interaction [17]. Liang et al. also showed that interaction between LIPSs and polymeric C_3_N_4_ (p-C_3_N_4_) exhibits strong ionic bonding, electrostatic, and vdW interactions which are beneficial for altering the bonding and spatial configuration of LIPSs which then modifies their redox kinetics [57]. Meng et al. also reported that the abundant nitrogen species on the surface of C_3_N_4_ nanosheet is able to facilitate LiPSs anchoring by a surface chemical adsorption mechanism due to the formation of a Li-N bond [58]. Wang et al. reported a first-principle study on the application of C_5_N as a LIPSs host. The isosurfaces of charge density difference presented in Fig. 7g showed that it exhibits an effective physical/chemical adsorption property, which enables LIPSs anchoring and charge transfer to its surface [27]. Carbon nitrides have also been explored for metal-air batteries because of their unique properties. For example, Shinde et al. reported the prospect of C_2_N for zinc-air (Zn-Air) batteries and showed that the open holey structure of C_2_N enabled reversible oxygen reactions and improved the electronic conductivity [59]. Je et al. also carried out first-principle calculations on heptazine and triazine sourced C_3_N_4_ for non-aqueous Li-O_2_ battery, and the results showed that triazine was better because it delivered a higher overpotential. Interaction of C_3_N_4_ with LiO_2_ resulted in the formation of a Li-N bonding, which is dependent on the ratio of N in the material and greatly influences their overpotential [60].

#### Doped Carbon Nitrides

**Fig. 8:**
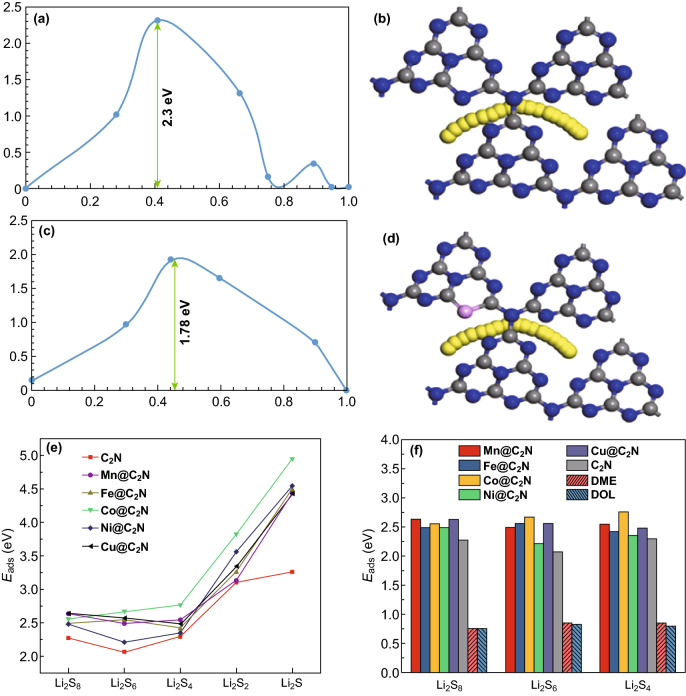
**a** Energy barrier for Na diffusion through g-C_3_N_4_, **b** corresponding Na diffusion path. **c** Energy barriers for Na diffusion through P-g-C_3_N_4_, **d** corresponding Na diffusion path. Reproduced with permission from Ref. [32]. **e** Adsorption energies of LiPSs on transition metal embedded C_2_N monolayers. **f** Adsorption energies of long-chain LiPSs with transition metal embedded C_2_N, 1,2-dimethoxyethane (DME) and 1,3-dioxolane (DOL). Reproduced with permission from Ref. [28]. Copyright permissions from Springer Nature and Elsevier. (Color figure online)

Theoretical study on doped carbon nitrides of different atomic compositions has been reported. For example, Nong et al. considered the effect of non-metal doping (boron, oxygen, and sulfur) and the impact of strain on the Li storage capacity and Li absorptivity of C_3_N. They reported that oxygen-doped C_3_N (O_N_–C_3_N) was the preferred dopant, it operated at 0.02 V and delivered a higher theoretical capacity of 534.42 mAh g^−1^ although it exhibited a high Li diffusion barrier of 0.78 eV, and this could be lowered by applying strain to the C_3_N structure [61]. Tian et al. also studied the effect of boron doping on the performance of C_3_N for alkali metal ion battery (Li, Na, and K) and showed that B_4_-doped C_3_N displays superior capacity, cycle, and thermal stability than pristine C_3_N [29]. Cha et al. showed that CN possesses a high Na adsorption energy, which can foster attraction of Na atoms into its structure for SIBs batteries [31]. Although Weng et al. [62] proved that C_3_N_4_ displays a very high Na-adsorption energy which limits its application for SIBs, the report of Molaei et al. on P-doped C_3_N_4_ showed that phosphorus doping is an effective way to decrease the high Na adsorption energy and diffusion barrier in C_3_N_4_ (Fig. 8a-d). The more P-atoms introduced into the structure of C_3_N_4_, the lower these energies [32]. Moreover, the work of Cha et al. [31] on Si-doped CN suggests that S-doping of carbon nitrides can successfully enlarge their interlayer distance for such large-sized metal-ion battery applications.

Doped carbon nitrides have also been studied for Li–S battery, Lin et al. studied the effect of transition metal doping (Mn, Fe, Co, Ni, and Cu) in C_2_N for improved immobilization of polysulfides in Li–S batteries, their results showed that a Lewis acid–base interaction which boosted LiPSs anchoring was established, and a significant charge was transferred from the transition metal dopant to the C_2_N monolayer with Co@C_2_N transferring the most in line with its superior adsorption energy (Fig. 8e, f) [28]. Zhao and co-workers reported confined single-atom Pt in holey g-C_3_N_4_, and DFT calculations showed that incorporation of single-atom Pt in holey g-C_3_N_4_ leads to improved electrical conductivity and a more stable structure with efficient electron and ionic transfer [63].

#### CNBCs

**Fig. 9:**
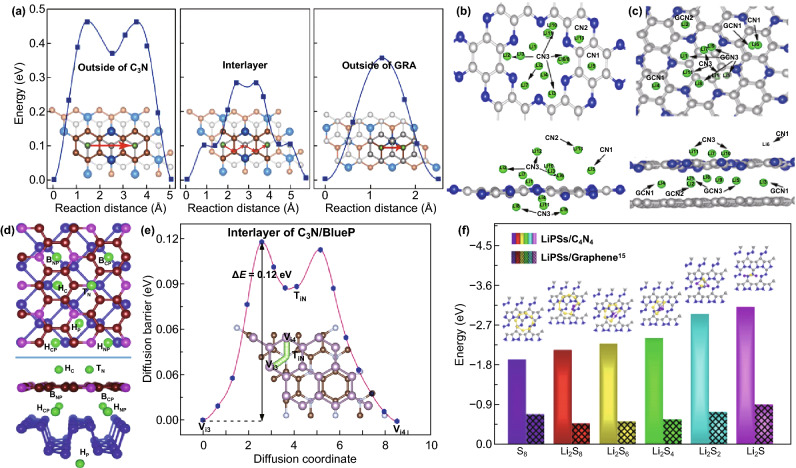
**a** Energy profiles for Li atom diffusion on C_3_N/GRA, along with the corresponding pathways denoted as red arrows. Carbon atoms from graphene—gray balls, carbon atoms from C_3_N—orange balls, nitrogen atoms—blue and lithium atoms—green. Reproduced with permission from Ref. [37]. **b** Top and side views of Li_13_–C_2_N structure; **c** top and side views of the Li_11_–C_2_N/graphene bilayer. Different adsorption sites are indicated as CN1, CN2, CN3, GCN1, GCN2, and GCN3 for both structures. Reproduced with permission from Ref. [38]. **d** Top and side views of the Li adsorption site on the C_3_N/P heterostructure (Li/C_3_N/P, C_3_N/Li/P, and C_3_N/P/Li). HC and TN sites are on the outer surface of C_3_N, and HP site is on the outer surface of phosphorene, HCP, HNP, BCP, and BNP sites are in the interlayer of the C_3_N/P heterostructure. Reproduced with permission from Ref. [64]. **e** Lithium migration pathway and corresponding energy profile through Path II of the interlayer of C_3_N/Blue P heterostructure. Reproduced with permission from Ref. [65]. **f** Adsorption energy for S_8_ cluster and LiPSs on C_4_N_4_ monolayer (bars without patterns) and graphene (bars with patterns), respectively. The insets on the pillars show S_8_ or Li_2_Sn/C_4_N_4_ structures generated by the principle of minimum energy. Reproduced with permission from Ref. [56]. Copyright permissions from Elsevier, American Chemical Society and Royal Society of Chemistry. (Color figure online)

CNBCs with superior electronic conductivity and higher capacity have also been studied through DFT. For example, Wang et al. reported a DFT study of C_3_N/graphene heterostructure for LIBs and proved that the electrical conductivity and structural stability can be significantly improved. The heterostructure recorded a high theoretical capacity of 1079 mAh g^−1^, a low Li diffusion barrier of 0.28 eV at the interlayer (Fig. 9a), and operated at a low open-circuit voltage of 0.13 V [37]. Ding et al. reported the first-principle study of C_2_N/graphene bilayer for LIBs, and by using molecular dynamic (MD) simulations, they predicted that Li storage follows a two-step process, i.e., migration through the *z*-direction via the large hole in the center of the C_2_N structure and on to the surface of the C_2_N membrane. The structures of different compositions of the bilayer heterostructures are presented in Fig. 9b, c. [38]. Guo et al. [64] and Lin et al. [65] also reported a DFT study on C_3_N/phosphorene heterostructure for LIBs, while Bao et al. explored its potential for Li/Na battery. The three reports concluded that the design of such C_3_N/phosphorene heterostructure would not only address the issues of phosphorene but also alleviate the problems of C_3_N for Li/Na storage. Storage of Li-ions occurred at the outer surface and interlayer of the heterostructure (Fig. 9d, e) [66].

DFT reports on CNBCs for Li–S batteries have also been widely reported. For instance, Liao and co-workers showed that the anchoring ability of C_3_N_4_ could be exploited in CNBCs [67]. Fan et al. also reported a carbon black blended g-C_3_N_4_ (g-C-coated) for Li–S battery and showed that two bonds (C-S and N-Li) are formed as a result of its interaction with LIPSs. These bonds inhibit the migration of LiPSs because it effectively binds to the LiPSs [68]. The work of Li et al. on porous C_4_N_4_ monolayer and C_4_N_4_/graphene composite for Li–S battery reported that LIPSs adsorption occurred by a chemisorption process and addition of graphene enhanced this process (Fig. 9f) [56]. Chen et al. studied the effect of transition metal (Fe, Ni, Cu, and Co)-doped g-C_3_N_4_/C composite for Li–S battery and showed that this would increase the electron mobility, while g-C_3_N_4_ will prevent polysulfide dissolution by anchoring LiPSs species [69]. In summary, DFT has been very key in the synthesis and electrochemical study of carbon nitrides for metal-ion batteries. Table 1 compares the DFT predicted parameters of carbon nitrides with commercial graphite anode for energy storage devices. From this table, it is obvious that most of the carbon nitrides can function as anode materials with superior theoretical capacity compared to graphite. These electrochemical properties have inspired researchers into the synthesis and fabrication of pure and doped carbon nitrides as well as CNBCs for energy storage devices.

**Table 1:** Comparison of the DFT predicted parameters of pure carbon nitrides with commercial graphite for energy storage devices

Materials	Conductivity	Capacity (mAh g^−1^)	Adsorption energy (eV)	Diffusion barrier (eV)	Operating voltage (V)
Graphite	Metallic	372 (LIBs) 31 (SIBs) [70] 279 (PIBs) [71]	1.1–1.29 (LIBs) [72] − 0.67 (SIBs) [73] − 0.44 to 2.0 (PIBs) [74]	0.22 (LIBs) [75] 0.09–0.35 (SIBs) [76] 0.039 (PIBs) [77]	0–0.5 (LIBs) [78] 0.3 (SIBs) [79] ~ 0.24 (PIBs) [80]
CN	Semi-conductor	454 (LIBs) [48]	– 5.03 (LIBs) [48]	~ 3 (LIBs) [45]	~ 0.6 (LIBs) [81]
C_2_N	Semi-conductor	2939 (LIBs) [49] 2469 (SIBs) [49]	– 3.437 (LIBs) [49] – 2.868 (SIBs) [49]	0.409 (LIBs) [49] 0.116 (SIBs) [49]	0.452 (LIBs) [49] 0.458 (SIBs) [49]
C_3_N	Semi-conductor	837.06 (LIBs) [40] 1072 (SIBs) [55] 1072 (PIBs) [55]	− 0.01 (LIBs) [40] − 1.806 (SIBs) [55] – 2.230 (PIBs) [55]	0.8 (LIBs) [40] 0.03 (SIBs) [55] 0.07 (PIBs) [55]	0.15 (LIBs) [40] 0.13 (SIBs) [55] 0.26 (PIBs) [55]
C_3_N_4_	Semi-conductor	524 (LIBs) [44]	– 4.56 (LIBs) [45]	~1.8 (LIBs) [47]	0.8 (LIBs) [44]
C_4_N	Semi-conductor	I—1942 (LIBs) [52] II -2158 (LIBs) [52]	I—1.3 (LIBs) II—0.93 (LIBs) [52]	I—0.26 [52] II—0.21 [52]	I—0.60 [52] II—0.68 [52]
C_5_N	Metallic	N/A	N/A	N/A	N/A

## Synthesis Strategies of Pure and Doped Carbon Nitrides

The conclusions obtained from DFT studies showed that the poor performance of most CNBMs can be traced to high metal atom adsorption energy due to excess graphitic-N, which leads to irreversible intercalation/deintercalation, poor conductivity, and low charge transfer mobility, as well as inferior structural stability after metal atom adsorption. DFT calculations showed that these problems could be overcome by regulating the ratio of C/N in the structure and prioritizing pyridinic-N, heteroatom doping, and CNBCs design with other superior electrode materials. Inspired by these DFT conclusions, several researchers have modified the general experimental synthesis strategies to obtain CNMBs that exhibit the desired structural/electronic properties. Such modified experimental synthesis strategies that are motivated by the findings in DFT studies can be considered DFT-guided synthetic protocols. In this section, we summarize some significant DFT-guided synthesis strategies for CNBMs, including pure/doped carbon nitrides as well as CNBCs.

### Top-Down Strategy

**Fig. 10:**
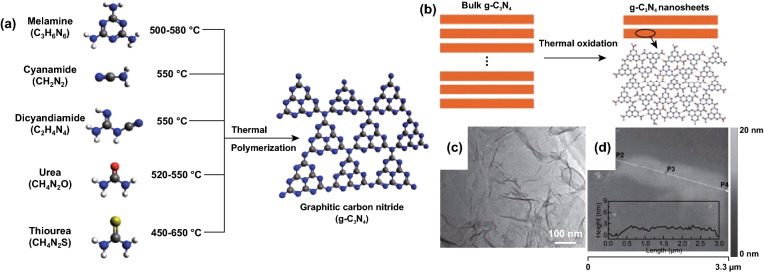
**a** Schematic illustration of one of the top-down synthesis approach (thermal polymerization) for g-C_3_N_4_ using different precursors. Black balls—carbon (C), blue balls—nitrogen (N), white balls—hydrogen (H), red balls—oxygen (O), and yellow balls—sulfur (S), respectively. Reproduced with permission from Ref. [84]. **b** Schematic of the synthesis of g-C_3_N_4_ nanosheets from bulk g-C_3_N_4_. In the atomic model, carbon atoms—gray balls, nitrogen atoms—blue balls and hydrogen atoms—red. **c** TEM image of g-C_3_N_4_ nanosheets. d Tapping-mode AFM image of a single g-C_3_N_4_ nanosheet deposited on the silicon wafer substrate. The inset is the height curve determined along the line between P1 and P4. Reproduced with permission from Ref. [88]. Copyright permissions from American Chemical Society and Wiley–VCH. (Color figure online)

Carbon nitrides generally occur in the bulk state, and this form of carbon nitride is obtained via thermal decomposition of N-rich amine compounds such as melamine [82] (Fig. 10a). However, bulk carbon nitride suffers from the poor conductivity, limited surface area, and sluggish reaction kinetics [83, 84]. Bulk carbon nitride also contains excess graphitic-N which is proven to limit the performance of carbon nitrides in rechargeable batteries according to DFT conclusions. One way to regulate the C/N ratio and decrease the graphitic-N is by converting bulk carbon nitride to nanosheets and other morphologies. This can be achieved through the top-down strategy which is grouped into post-thermal oxidative etching and ultrasonic liquid exfoliation.

#### Thermal Oxidation

As the name implies, this method involves the heat treatment of bulk carbon nitride at high temperature and it is an effective way to break the van der Waals forces which hold the layer of carbon nitride together. This process produces a sheet-like carbon nitride with large surface area, surface defects and adjusted layer thickness [22, 85]. As depicted in Fig. 10b, bulk g-C_3_N_4_ can be converted to g-C_3_N_4_ nanosheets via thermal oxidation and Fig. 10c, d shows that the layer thickness can also be regulated. Guo et al. reported the synthesis of graphite/sheet-like carbon nitride (CN) as well as nanotube, nanoribbon, and microsphere morphologies via thermal treatment [86, 87]. 2D-C_3_N carbon nitride with a needle-like morphology was also synthesized by via a direct solid-state reaction [23].

#### Ultrasonic Liquid Exfoliation

**Fig. 11:**
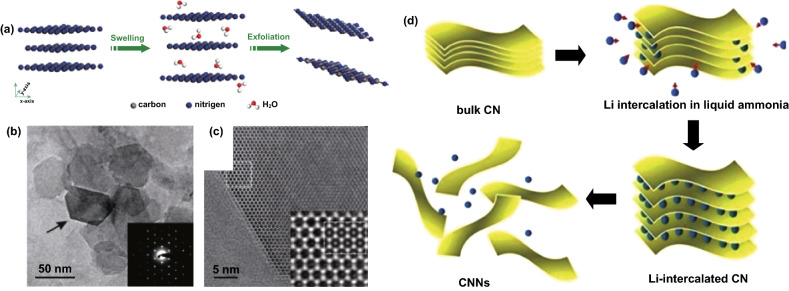
**a** Schematic illustration of liquid-exfoliation process from bulk g-C_3_N_4_ to ultrathin nanosheets. Reproduced with permission from Ref. [90]. **b** TEM image of exfoliated ultrathin nanosheets, **c** higher magnification of a carbon nitride nanosheet edge viewed along [001]. Reproduced with permission from Ref. [91] **d** Schematic diagram of the lithiation and exfoliation of g-C_3_N_4_ nanosheets from bulk g-C_3_N_4_. Reproduced with permission from Ref. [92]. Copyright permissions from American Chemical Society. (Color figure online)

Another effective strategy to convert bulk multilayered carbon nitrides to few/single layer carbon nitrides is through the intercalation of solvent molecules through the bulk structure, thereby resulting in delamination (Fig. 11a). This liquid exfoliation process is effective for synthesizing nanosheets or flakes of carbon nitrides (Fig. 11b, c). Mahmood et al. reported the synthesis of two-dimensional C_2_N carbon nitride by a top-down (facile wet-chemical reaction) approach [89]. This process can also be achieved through lithiation exfoliation which uses Li ions as exfoliant to penetrate the layers of bulk carbon nitride and separate them to individual nanosheets (Fig. 11d).

### Bottom-Up Strategy

The bottom-up strategy or template-aided synthesis is divided into soft and hard templating method.

#### Hard Templating

**Fig. 12:**
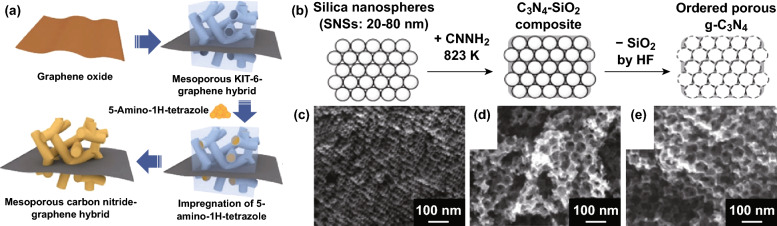
**a** Schematic representation of the synthesis protocol for synthesizing the mesoporous C_3_N_5_ and its graphene hybrid. Reproduced with permission from Ref. [98]. **b** Schematic illustration of the synthesis of ordered porous g-C_3_N_4_ by using close-packed silica nanospheres (SNSs) as the primary template. **c**–**e** FE-SEM images of porous g-C_3_N_4_ at different resolutions. Reproduced with permission from Ref. [93]. Copyright permissions from Wiley–VCH and Royal Society of Chemistry. (Color figure online)

Templates are often used as morphology directing agents during the synthesis of unique carbon nitride morphologies. Hard templates such as silica [93–95], anodic alumina oxide [96], and carbon [97] are often reported for synthesizing CNBMs. For instance, DFT studies showed that by increasing the N content in C_3_N_4_ to C_3_N_5_ the electrochemical properties can be improved. Recently, Kim et al. reported the synthesis of a graphene-like mesoporous carbon nitride C_3_N_5_ with superior electronic properties by using KIT-6 as a hard template [98]. Moreover, their DFT/electrochemical study showed that such composite can deliver excellent performance for energy storage devices [34]. The C_3_N_5_ sample showed outstanding interesting electrochemical properties, and it was combined with graphene oxide to form a composite (MCN-11). Figure 12a shows a scheme of the synthesis procedure of the C_3_N_5_ and the hybrid.

#### Soft Templating

Templating materials which demonstrate self-assembling properties have also been employed for synthesizing carbon nitrides by using ionic liquids [99, 100], surfactants [101–103], and amphiphilic block polymers [102, 104]. Yong et al. reported the synthesis of a nanoporous carbon nitride by using ionic liquid as soft templates (Fig. 12b-e), and such carbon nitride structure demonstrated superior conductivity to bulk material [101]. In summary, by using the conclusions of DFT studies as a guide, the general experimental synthesis approach of carbon nitrides can be optimized to achieve carbon nitrides with regulated C/N ratio, increased pyridinic-N, superior structural stability, and conductivity for improved performance.

### Fabrication Strategies of Doped Carbon Nitrides

**Fig. 13:**
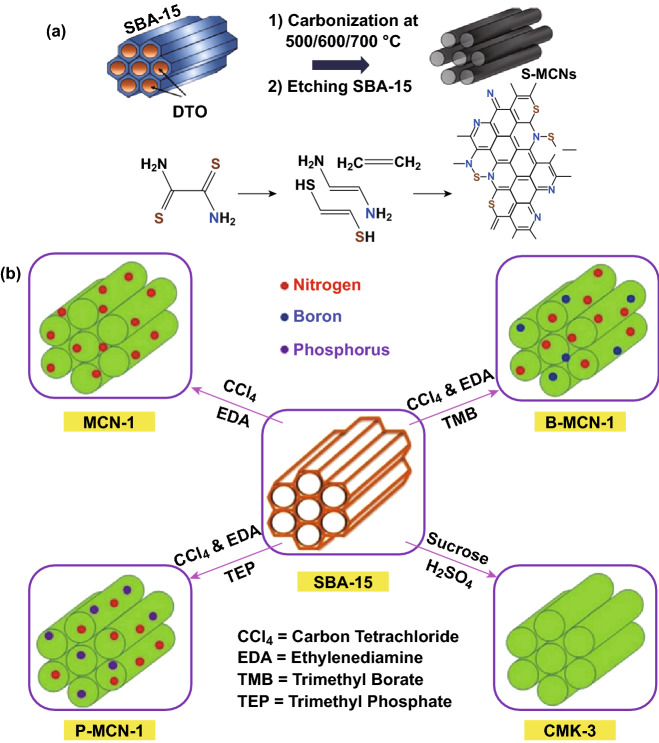
**a** Schematic of the synthesis procedure of S-MCN [31]. **b** Schematic of the template synthesis method for heteroatom-doped mesoporous carbon nitrides. Reproduced with permission from Ref. [105]. Copyright permission from American Chemical Society. (Color figure online)

The DFT study of Molaei et al. [32] proved that heteroatom doping of carbon nitride will boost the electronic conductivity and improve metal atom storage, and the DFT conclusion of this work motivated the Vinu group to report the synthesis of rod-like sulfur-doped mesoporous CN (S-CN) by a templating method (Fig. 13a) [31]. Phosphorus-doped mesoporous carbon nitride (P-MCN) was also synthesized via a simple template approach [105]. A schematic of the template synthesis method for heteroatom-doped mesoporous carbon nitrides is presented in Fig. 13b. To summarize, doped carbon nitrides can be synthesized through a combination of some top-down and bottom-up approaches which often involves the use of a salt of the heteroatom or a surfactant.

## Fabrication Strategies for CNBCs

DFT studies have proved that CNBCs design is another effective way to resolve the challenges of carbon nitrides because such CNBCs will demonstrate superior conductivity, better structural stability, and enhanced charge transfer. Therefore, in this section, we highlight some recent CNBCs that have been synthesized through commonly reported experimental synthesis strategies but inspired by the conclusions of DFT studies.

### Hydrothermal Method

**Fig. 14:**
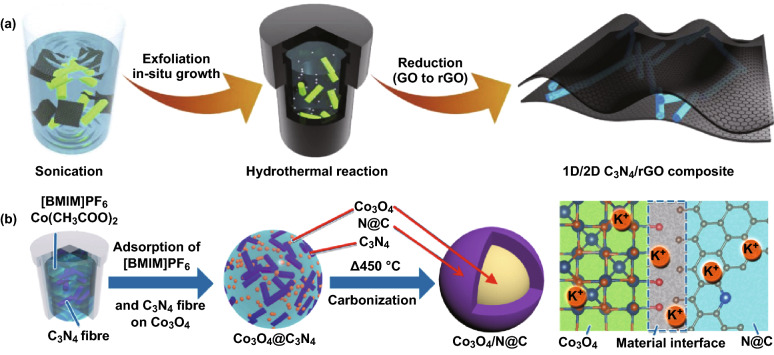
**a** Schematic illustration of the synthesis of 1D/2D C_3_N_4_/rGO composite via a hydrothermal/freeze-drying method. Reproduced with permission from Ref. [54]. **b** Scheme illustrating suggested potassiation and depotassiation mechanism of the Co_3_O_4_@N–C electrode. Reproduced with permission from Ref. [108]. Copyright permission from Elsevier and American Chemical Society. (Color figure online)

Hydrothermal method involves the controlled synthesis of a composite in a tightly sealed vessel under high temperature and pressure [106, 107]. Although 2D-C_3_N_4_ is widely reported for designing CNBCs, our DFT studies showed that 1D-C_3_N_4_ exhibits superior structural stability and metal storage capability than 2D-C_3_N_4_ [40]. Moreover, our comparative DFT study between 1D/2D C_3_N_4_/graphene and 1D/2D C_3_N_4_/graphene showed that 1D/2D will perform better [38]. These DFT conclusions motivated us to combine 1D-C_3_N_4_ with 2D-rGO for energy storage devices. Therefore, we reported the synthesis of a 1D/2D C_3_N_4_/rGO composite via a freeze-drying-assisted hydrothermal approach by deploying the *π*–*π* interaction between C_3_N_4_ and graphene [54]. Figure 14a shows the synthesis scheme of the composite. Guided by the result of our DFT calculations, we also reported the design of a composite of Co_3_O_4_@N–C derived from 1D-C_3_N_4_ via an ionic liquid-assisted solvothermal method [108]. Scheme illustration of the synthesis strategy is depicted in Fig. 14b.

### Self-Assembly Method

**Fig. 15:**
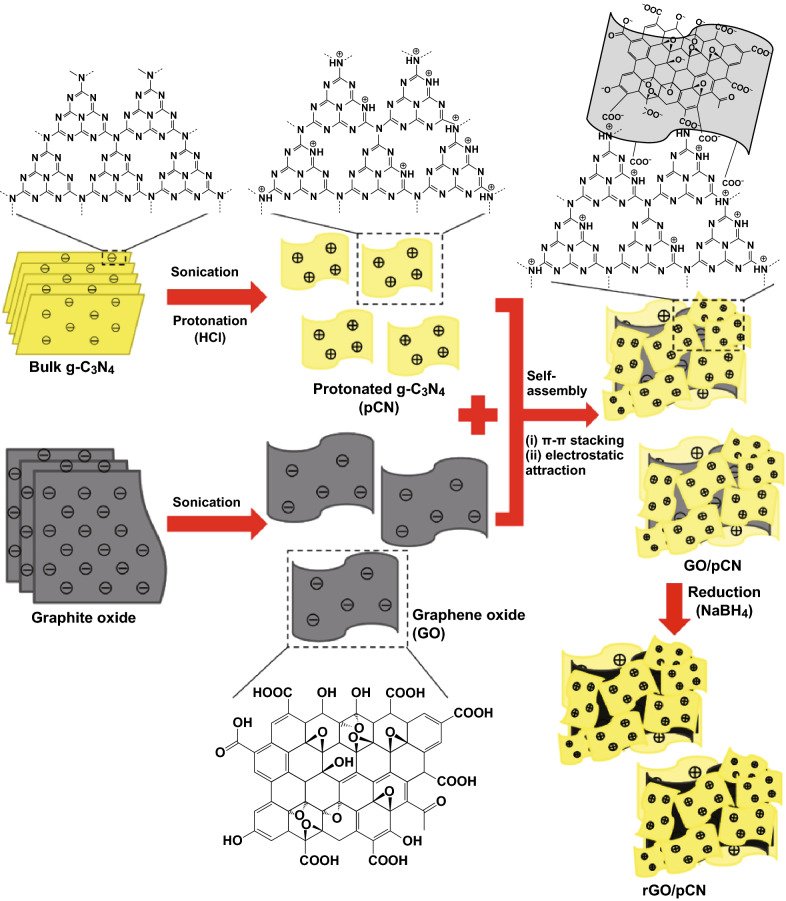
Schematic diagram for the synthesis process of rGO/pCN samples via a combined ultrasonic dispersion and electrostatic self-assembly strategy followed by a NaBH_4_-reduction process. Reproduced with permission from Ref. [109]. Copyright permission from Elsevier. (Color figure online)

Due to their amphoteric nature, carbon nitrides possess tunable surface functional groups and surface charges which enable surface attachment with other functional materials such as graphene and other functional 2D materials [130]. Such structural interaction has been identified through the molecular dynamic simulation and DFT studies of Ding et al. and Wang et al. The heterostructure will display superior electronic conductivity and structural stability than the pure carbon nitride. This concept is employed in self-assembly synthesis of C_3_N_4_ and other CNBMs. Figure 15 shows a detailed schematic of the self-assembly approach for a surface modified C_3_N_4_ (pCN) with graphene oxide and reduced graphene oxide to achieve a GO/pCN and rGO/pCN composite, respectively [109].

### Other Composite Fabrication Strategies

**Fig. 16:**
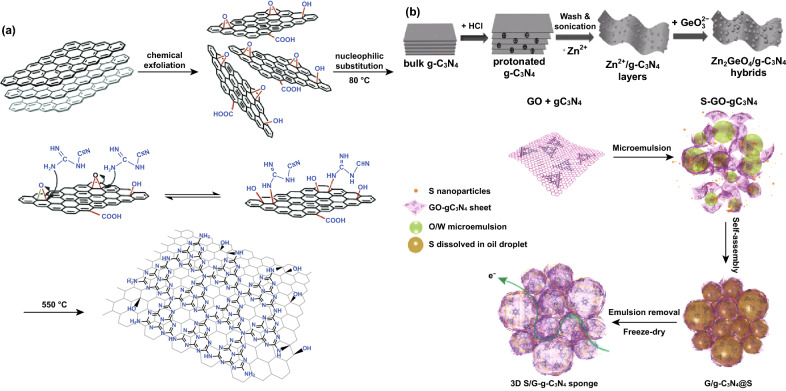
**a** Illustration of the formation process of g-C_3_N_4_–rGO. Reproduced with permission from Ref. [110]. **b** Schematic of the synthesis process of the Zn_2_GeO_4_/g-C_3_N_4_ hybrids. Reproduced with permission from Ref. [111]. c Schematic illustration of the procedure for preparing S/GCN hybrid sponge. Reproduced with permission from Ref. [113]. Copyright permissions from Royal Society of Chemistry and Wiley–VCH. (Color figure online)

CNBCs can also be designed by some other unique methods, and Fu et al. designed a 2D-2D g-C_3_N_4_-rGO hybrid via an in situ chemical method by initiating a nucleophilic reaction between epoxy groups on the surface of GO and amine/amide groups on dicyanamide-C_2_H_4_N_4_. The synthesis method is depicted in Fig. 16a, and through this approach, the problem of poor electrical conductivity, aggregation, and restacking was resolved [110]. Similarly, Li et al. reported the design of a Zn_2_GeO_4_/g-C_3_N_4_ composite by growing Zn_2_GeO_4_ NPs in-between the layers of g-C_3_N_4_ via a solution approach to inhibit agglomeration of Zn_2_GeO_4_ NPs and restacking of g-C_3_N_4_ (Fig. 16b). Zn_2_GeO_4_ also functioned as a spacer to enlarge the interlayer distance of g-C_3_N_4_ sheet enabling metal ion adsorption and improved conductivity [111]. Wang et al. reported the synthesis of a GO/g-C_3_N_4_ microsphere by an ethanol-assisted spray drying approach [112]. Zhang et al. also reported the design of a sponge-like free-standing 3D S/graphene@g-C_3_N_4_ hybrid using a microemulsion-assisted assembly method (Fig. 16c) [113].

In summary, the advancement in experimental synthesis strategies of pure/doped carbon nitrides and CNBCs has been propelled by the conclusions of DFT studies on CNBMs. Precisely, DFT studies proposed that adjusting the C/N ratio, increasing the concentration of pyridinic-N, heteroatom doping and CNBCs design are effective ways to inhibit the problem of irreversible intercalation/deintercalation, poor conductivity, and instability experienced by pure layered CNBMs in rechargeable batteries. These conclusions from DFT studies have guided the experimental synthesis of CNBMs to fabricate functional CNBMs which exhibit the desired requirements and deliver superior battery performance.

## Electrochemical Studies of CNBMs for Energy Storage Devices

The structural/electronic properties and surface functionalities of CNBMs qualify them as promising electrode materials for energy storage devices. In this section, we give an overview of experimental works on carbon nitrides for energy storage devices including LIBs, SIBs and PIBs, Li–S, LABs, LMBs, ZABs, and SSBs.

### Lithium-Ion Batteries (LIBs)

LIBs offer benefits such as lightweight, superior energy density, and long cycle life [3, 114, 115]; CNBMs (pure/doped carbon nitrides and CNBCs) have been reported as electrodes.

#### Pure Carbon Nitrides for LIBs

**Fig. 17:**
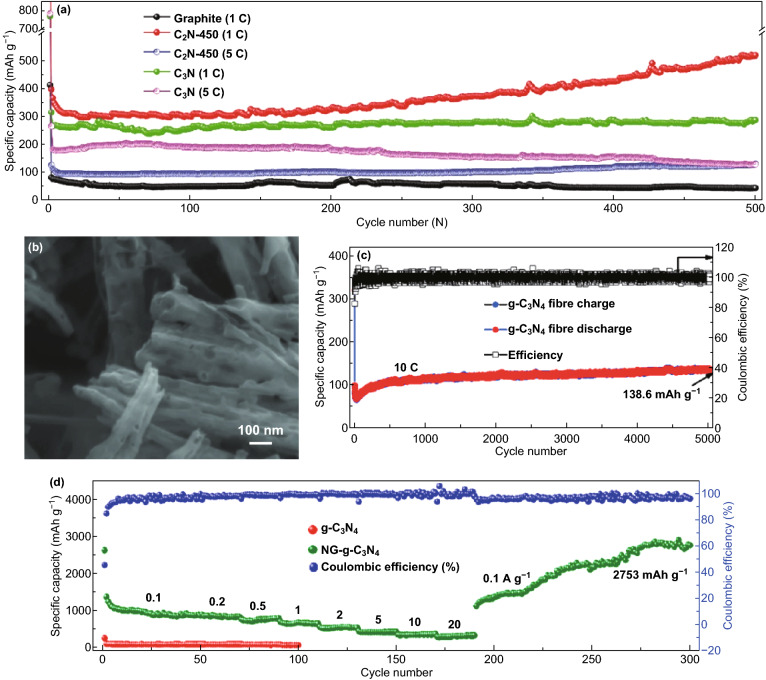
**a** Long cycle life of C_2_N-450, C_3_N. Reproduced with permission from Ref. [50]. **b** SEM image of the 1D-g-C_3_N_4_ fiber. **c** Cycling performance of 1D-g-C_3_N_4_ fiber structure at a high current density of 10 C. Reproduced with permission from Ref. [46]. **d** Rate performance for ND-g-C_3_N_4_ electrode at current density of 0.1, 0.2, 0.5, 1, 2, 5, 10, and 20 A g^−1^ and galvanostatic discharge property for g-C_3_N_4_ electrode. Reproduced with permission from Ref. [8]. Copyright permissions from Wiley–VCH, and American Chemical Society. (Color figure online)

Due to the structural and electronic properties of carbon nitrides, different atomic composition of carbon nitrides has been studied for LIBs. Yin and co-workers reported C_3_N_3_ for LIBs but only a low reversible capacity of 197.8 mAh g^−1^ was obtained at 100 mA g^−1^ after 300 cycles and a ICE of 34.3% [81]. Xu et al. tested C_2_N (C_2_N-450) and C_3_N for LIBs and at 1C, C_3_N only retained 285.1 mAh g^−1^ after 500 cycles, while C_2_N‐450 maintained a reversible capacity of 516.1 mAh g^−1^ although it displayed large activation (Fig. 17a) [50]. Other carbon nitride compositions such as C_4_N and C_5_N have not been experimentally tested for any metal ion batteries.

We reported the synthesis of a porous 1D-C_3_N_4_ fiber with large surface area and multiple active sites (see SEM image—Fig. 17b) for LIBs; a reversible capacity of 181.7 mAh g^−1^ was achieved at 0.5 C and 138.6 mAh g^−1^ at 10 C (Fig. 17c) [46]. Chen et al. also reported the application of C_3_N_4_ (ND-g-C_3_N_4_) for LIBs and obtained a capacity of 2753 mAh g^−1^ after 300 cycles) although significant activation occurred (Fig. 17d). To provide a brief summary, pure carbon nitrides of different crystalline structures have been reported for LIBs, and commendable performances have been recorded (Table 2). However, the issues of ineffective intercalation/deintercalation due to extremely high Li adsorption energy, high Li diffusion barrier, structural deformation, and loss of crystallinity has severely limited the exploitation of their full potential. Therefore, DCNs and CNBCs are necessary to achieve improved performance.

**Table 2:** Pure and doped carbon nitrides for LIBs and SIBs

Battery type	Materials	Synthesis	Morphology	Initial capacity (mAh g^−1^)	ICE^*a*^	Reversible capacity (mAh g^−1^)	References
LIBs	ND-g-C_3_N_4_	Magnesiothermic denitriding method	Porous nanosheet	2627 @ 100 mA/g	45.7%	2753 @ 300 cycles	[8]
	CN-480–600	Solid-state Wurtz reaction	Macroporous graphite-like	575.7 @ 100 mA/g	19.9%	197.8 @ 300 cycles	[144]
	C_2_N-450	Bottom-up wet-chemical reaction.	Stacked sheet	1629.6 @ 0.1 C	57.2%	516.1 @ 500 cycles	[146]
	C_3_N	Bottom-up wet-chemical reaction.	Rod-like	787.3 @ 0.1 C	48.7%	1C, 285.1 @ 500 cycles	[126]
	Li-C_3_N_4_	Electrochemical and solid-state reactions	Sheet-like	188	–	38 @ 6 cycles	[121]
	C_6_N_8_ (C_3_N_4_)	Thermal oxidation	–	250 @ 30 mA/g	–	50 @ 50 cycles	[122]
	1D-C_3_N_4_ fiber	Polyaddition/polycondensation reaction	Porous layered fiber	419.7 @ 0.5 C	84.1%	181.7 @ 200 cycles	[46]
	NGC	Carbonization	Graphene-like	2749 @ 50 mA/g	50.3%	1143 @ 200 cycles	[145]
	P-MCN-1	Template synthesis	Spherical particles	2850 @ 1 A/g	44.2%	963 @ 1000 cycles	[105]
SIBs	C_6_N_8_ (C_3_N_4_)	Thermal oxidation	–	250 @ 30 mA/g	–	10 @ 50 cycles	[122]
SIBs	S-MCN	Hard template approach	Rod-like morphology	~850 @ 100 mA/g	47%	304.2 @ 100 cycles	[31]

^a^*ICE* Initial coulombic efficiency

#### CNBCs for LIBs

CNBCs can deliver superior structural/electronic properties, enhanced structural stability and extremely large surface area which will boost the overall LIBs performance. In this section, we highlight some significant CNBCs including binary CNBCs and ternary CNBCs.

##### Binary CNBCs for LIBs

**Fig. 18:**
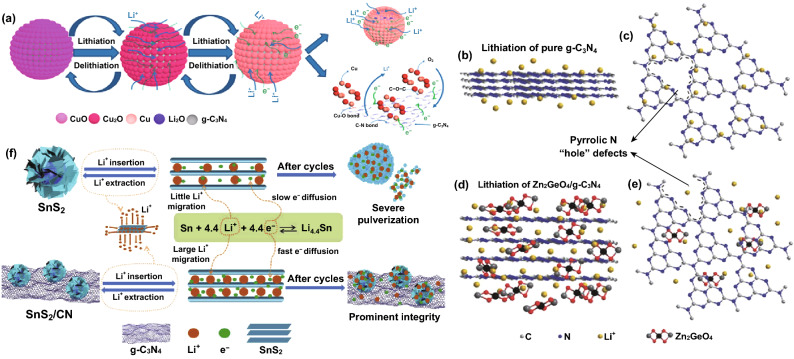
**a** Schematic description explaining the reaction mechanism of the CuO/O-doped g-C_3_N_4_ anode during the charge/discharge process. Reproduced with permission from Ref. [116]. Illustrations of pure (**b**, **c**) g-C_3_N_4_ and (**d**, **e**) Zn_2_GeO_4_/g-C_3_N_4_ hybrids for Li-insertion viewed from the (**b**, **c**) edge and (**d**, **e**) basal plan directions. Reproduced with permission from Ref. [111]. **f** Schematic illustration of the Li-ions diffusion and electronic transport in the SnS_2_ and SnS_2_/CN composite electrode during the charge/discharge processes. Reproduced with permission from Ref. [117]. Copyright permissions from Elsevier, Royal Society of Chemistry, American Chemical Society and Wiley–VCH. (Color figure online)

Binary CNBCs involving carbonaceous materials such as graphene oxide are commonly reported because of the inherent *π*–*π* stacking, extremely large surface area, surface functional groups, and electronic conductivity. For instance, Fu et al. [110] reported the application of a 2D-2D stacked g-C_3_N_4_-rGO hybrid for LIBs. The 2D-2D stacked g-C_3_N_4_-rGO hybrid achieved a reversible capacity of 1525 mAh g^−1^ at 100 mA g^−1^ and up to 943 mAh g^−1^ at 1000 mA g^−1^ [110]. Mohamed et al. designed a CuO/O-doped g-C_3_N_4_ composite which delivered a reversible capacity of 738 mAh g^−1^ after 100 cycles and 503 mAh g^−1^ after 500 cycles when tested at 100 and 1000 mA g^−1^, respectively. A depiction of the Li^+^ storage mechanism is shown in Fig. 18a [116]. Li et al. also reported the application of metal oxide-based composite—Zn_2_GeO_4_/g-C_3_N_4_ composite for LIBs. The lithium storage mechanism for g-C_3_N_4_ and the composite is depicted by the scheme provided in Fig. 18b-e. The composite exhibited a superior reversible capacity of 1370 mAh g^−1^ at 200 mA g^−1^ after 140 cycles [111]. Yin et al. designed a composite comprising of SnS_2_ anchored on g-C_3_N_4_ nanosheet, and the SnS_2_/CN composite followed the conversion ${\text{SnS}}_{2} + {\text{xLi}}^{ + } + {\text{xe}}^{ - } \to {\text{Li}}_{{\text{x}}} {\text{SnS}}_{2}$ and alloying/dealloying lithium storage mechanism (${\text{Sn}} + 4.4{\text{Li}}^{ + } + 4.4{\text{e}}^{ - } \to {\text{Li}}_{4.4} {\text{Sn}}$). Schematic of this storage mechanism is provided in Fig. 18f. A high reversible capacity of 444.7 mAh g^−1^ was delivered at 100 mA g^−1^ after 100 cycles with structural stability and no pulverization [117].

##### Ternary CNBCs for LIBs

**Fig. 19:**
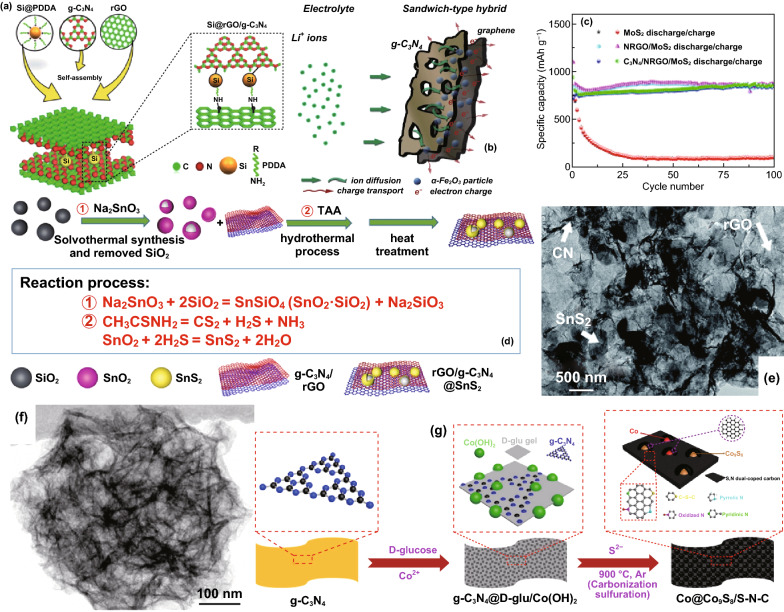
**a** Schematic reaction scheme of the Si@rGO/g-C_3_N_4_ hybrid and illustration of Si NPs anchored on rGO/g-C_3_N_4_ via strong covalent and hydrogen bonding formed during the pyrolysis process. Reproduced with permission from Ref. [118]. **b** Schematic diagram of ionic diffusion and charge transport in the porous Fe_2_O_3_/CN–G anode with a 2D sandwich-like nanosheet architecture. Reproduced with permission from Ref. [124]. **c** Cycle performance over the voltage range of 0.01–3.0 V vs. Li/Li^+^ at the same current density of 100 mA/g for MoS_2_, NRGO/MoS_2_, and C_3_N_4_/NRGO/MoS_2_. Reproduced with permission from Ref. [121]. **d** Schematic synthesis process of rGO/g-C_3_N_4_@SnS_2_. Reproduced with permission from Ref. [123]. **e** TEM micrograph of porous ternary composite architectures of reduced graphene oxide, SnS_2_, and CN (GSC6). Reproduced with permission from Ref. [122]. Copyright permissions from Royal Society of Chemistry, Elsevier, and Springer Nature. (Color figure online)

Ternary composites which can provide additional benefits than binary composites have also been reported. Wang et al. reported the application of a Si@rGO/g-C_3_N_4_ composite for LIBs. By taking advantage of the interfacial chemical bonding on functionalized rGO/g-C_3_N_4_, Si nanoparticles (NPS) were effectively anchored on 2D-composite of rGO/g-C_3_N_4_ (Fig. 19a), and such composite design resulted in a reversible capacity of 1354.8 and 799.6 mAh g^−1^ when cycled at 0.1 and 0.5 C, respectively [118]. Kong et al. also reported a red phosphorus/rGO-C_3_N_4_ composite which delivered a capacity of 1032.6 mA g^−1^ after 600 cycles when tested at 200 mA g^−1^ [119]. Shi et al. reported the synthesis of a Fe_2_O_3_/C_3_N_4_-graphene composite for LIBs. The Li storage mechanism of the Fe_2_O_3_/CN-G composite is depicted in Fig. 19b, a high reversible capacity of 980 mAh g^−1^ at 50 mA g^−1^ after 50 cycles along with a stable rate performance exceeding that of Fe_2_O_3_/G [120] (Table 3).

**Table 3:** CNBCs for metal-ion batteries LIBs, SIBs and PIBs

Battery type	Materials	Synthesis	Morphology	Initial capacity (mAh g^−1^)	ICE^*a*^	Reversible capacity (mAh g^−1^)	References
LIBs	huCP/g-C_3_N_4_	Hydrothermal treatment	Carbon fibers with large pores and hollow structure	1199 @ 1 A/g	~ 93%	1030 @ 1000 cycles	[147]
	g-C_3_N_4_–rGO	In-situ chemical synthetic approach	2D sheets	3002 @ 100 mA/g	57%	1525 @ 50 cycles	[108]
	CN-rGO	Hydrothermal synthesis	3D Sandwich architecture	1632 @ 50 mA/g	41.3%	970 @ 300 cycles	[130]
	g-C_3_N_4_@rGO	Hydrothermal reaction	Spongy, porous, and tangled ultrathin sheets	2731 @ 0.1 C	~89%	901 @ 50 cycles	[131]
	SN/CN	Hydrothermal	Cauliflower-like morphology	733 @ 0.1 C	55.4%	~420 @ 60 cycles	[132]
	SnO_2_@C_3_N_4_	Scalable solid-state reaction	Porous and wrinkled material	1200 @ 0.1 C	~50%	550 @ 100 cycles	[149]
	TiO_2_@CNS (TCNS)	Self-assembly approach/hydrothermal	Spherical Core–shell particles	359 @ 0.1 C	~90%	303 @ 125 cycles	[100]
	Zn_2_GeO_4_/g-C_3_N_4_	Facile solution approach	ultrathin nanosheet.	1068 @ 200 mA/g	58.6%	1370 @ 140 cycles	[109]
LIBs	NiCo_2_O_4_/g-C_3_N_4_	Facile ultrasonic treatment	Nanosheet structure	1367 @ 100 mA/g	84.5%	1252 @ 100 cycles	[150]
	Li_4_Ti_5_O_12_/g-C_3_N_4_	Solvothermal method	Nanoparticles	173.7 @ 0.5 C	–	150.8 @ 502 cycles	[133]
	Nitrogen-doped LTO/C (NCLTO)	Thermal decomposition	Irregular sized nanoparticles	189 @ 1 C	~92%	122 @ 500 cycles	[109]
	CuO/O-doped g-C_3_N_4_	Hydrothermal	Nanospheres	980 @ 100 mA/g	94.7%	738 @ 100 cycles	[101]
	3D N-rich C_3_N_4_@MoS_2_	Hydrothermal	Nanosphere	2390 @ 0.1 C	~74%	857 @ 50 cycles	[154]
	MoS_3_/g-C_3_N_4_–H^+^/GO	Sonication/thermal treatment	2D sheet-like structure	1728 @ 0.1 mA/g	–	1450 @ 200 cycles	[103]
	MoS_2_/g-C_3_N_4_	Thermal treatment (calcination)	Spherical particles	2467 @ 0.05 C	~41%	1204 @ 200 cycles	[175]
	WS_2_/g-C_3_N_4_	Solid-state reaction	Nano-sized petal-like sheets	1933.6 @ 100 mA/g	63.6%	622.7 @ 400 cycles	[156]
	SnS_2_/CN	Microwave hydrothermal method	Nanoflower	1465.9 @ 100 mA/g	47%	383.8	[153]
	Co_1−x_S@g-C_3_N_4_	Solvothermal method	Spherical-like	~1250 @ 0.1 A/g	~55%	789.59 @ 210 cycles	[155]
	C_3_N_5_/MoS_2_	Soft- and hard templating methods	Highly ordered mesoporous nanosheet	271 @ 100 mA/g	60%	193 @ 100 cycles	[112]
SIBs	huCP/g-C_3_N_4_	Hydrothermal treatment	Carbon fibers with large pores and hollow structure	222 @ 0.1 A/g	–	345 @ 380 cycles	[147]
	N-doped MoC	Pyrolysis	Hollow microspheres	1040 @0.16 A/g	–	410@ 200 cycles	[172]
	Amorphous carbon nitride (ACN)	Copolymerization/direct carbonization	Uniform rhombic dodecahedral shape	640 @ 83 mA/g	67.2%	175 @ 2000 cycles	[173]
	N-FLG-800	Pyrolysis	2D lamellar structure of with plenty of wrinkles	256.7 @ 0.5 A/g	83.5%	211.3 @ 2000 cycles	[175]
	C/g-C_3_N_4_	One-pot calcination	2D-sheet-like structure	~200 @ 0.4 A/g	99 %	~ 160 @ 400 cycles	[128]
	g-C_3_N_4_ film	Chemical vapor deposition (CVD)	2D thin film	–	98 %	~6 Ah/g @ 500 cycles	[174]
	CN/MoS_2_-600	Nanotemplating approach	Corrugated cardboard-like morphology	–	70%	605	[98]
	C_3_N_5_/MoS_2_	Combined soft- and hard templating methods	Ordered mesoporous nanosheet	126 @ 100 mA/g	46%	54 @ 100 cycles	[112]
PIBs	1D/2D C_3_N_4_/rGO	Hydrothermal/freeze drying method	1D-C_3_N_4_ rod infused in-between the 2D-sheet	682.7 @ 0.5 A/g	56.8%	557.4 @ 50 cycles	[54]
PIBs	Co_3_O_4_@N–C	Ionic liquid-assisted solvothermal method	Spherical	1229.2 @ 50 mA/g	48.2%	448.7 @ 40 cycles	[176]
LIBs	Si@rGO/g-C_3_N_4_	Template-free self-assembly and pyrolysis process	Multilayered 3D framework	1354.8 @ 0.5 C	70.9%	799.6 @ 1000 cycles	[159]
	P/rGO-C_3_N_4_	Ball milling	Nubby structure (40–200 nm)	2423.4 @ 200 mA/g	99%	1032.6 @ 600 cycles	[160]
	rGO/g-C_3_N_4_/SnS_2_ (GSC6)	Hydrothermal route	3D porous structure	1073.1 @ 100 mA/g	61.1%	1118.6 @ 100 cycles	[122]
	rGO/g-C_3_N_4_@SnS_2_	Solvothermal synthesis	Nano-spheres anchored on hybrid sheet	~600 @ 800 mA/g	——	864.9 @ 1000 cycles	[164]
	MnO/C_3_N_4_/C	Sonication/calcination	3D Porous sphere	918.9 @ 0.5 C	66%	781.9 @ 86 cycles	[165]
	SnO_2_-TiO_2_-C_3_N_4_	Self-assembly deposition	Microspherical shaped NPs	853 @ 0.1 C	——	114.1 @ 20 cycles	[188]
	Co@Co_9_S_8_/S–N-C	Hydrothermal reaction and pyrolysis method	Porous structure with smooth-faced surfaces	1033.25 @ 0.2 A/g	60%	652.1 @ 610 cycles	[166]

^a^ICE Initial coulombic efficiency

Ternary CNBCs involving metal chalcogenides such as MoS_2_ and SnS_2_ have also been studied. Hou et al. reported a C_3_N_4_/NRGO/MoS_2_ composite which exhibited a reversible capacity of 855 mAh g^−1^ after 100 cycles when tested at 100 mA g^−1^ (Fig. 19c) [121]. Shah et al. reported the design of a SnS_2_, rGO and g-C_3_N_4_ composite with intimate 2D-2D contact for LIBs. A stepwise detail of the synthesis method and the reaction processes involved is depicted in Fig. 19d. A reversible capacity of 1248.4 mAh g^−1^ was retained after 276 cycles at 100 mAg^−1^ [122]. Shi et al. also prepared a rGO/g-C_3_N_4_@SnS_2_ composite with effective surface contact of all constituents which can be seen from the TEM image (Fig. 19e). After 1000 cycles at 800 mA g^−1^, a reversible capacity of 864.9 mAh g^−1^ was retained [123].

### Sodium and Potassium-Ion Batteries (SIBs and PIBs)

**Fig. 20:**
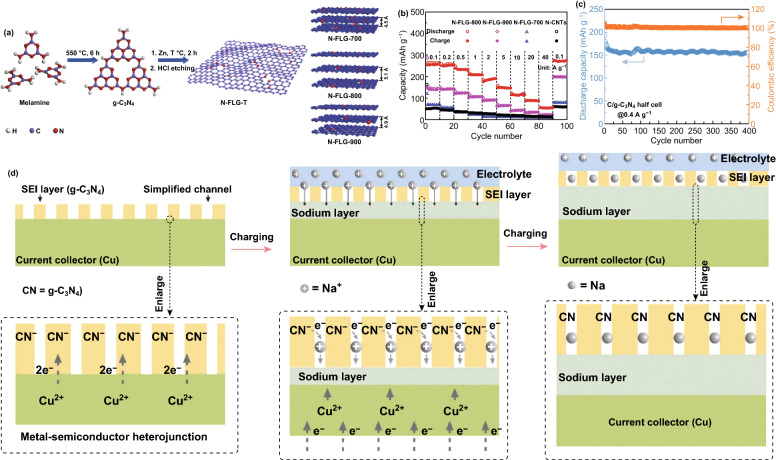
**a** Schematic illustration of the synthetic routes for the fabrication of N-FLG-T. **b** Rate capability of N–CNTs and N-FLG-T at various current densities. Reproduced with permission from Ref. [128]. **c** Cycling performance of C/g-C_3_N_4_ Na half cells at 0.4 A g^−1^. Reproduced with permission from Ref. [53]. d Illustration of the mechanism of the sodium storage in g-C_3_N_4_ film activated Cu foil. Reproduced with permission from Ref. [129]. Copyright permission from Wiley–VCH. (Color figure online)

Due to their large-sized ions, one of the major focus for sodium and potassium ion battery electrode materials is the enlargement of their interlayer distance. The low interlayer/*d*-spacing of graphite (0.334 nm) and its low theoretical capacity (279 mAh g^−1^) has limited its application in either of these two battery systems [125–127]. g-C_3_N_4_ can be a source of N-doped carbon with large interlayer distance; this approach was reported by Qiao et al. who successfully fabricated a series of N-doped graphene 2D-sheet with different interlayer distances (Fig. 20c). The optimized N-doped graphene sheet (N-FLG-800) delivered a superior rate capability of 56.6 mAh g^−1^ at a current density of 40 A g^−1^ (Fig. 20e) and outstanding long-cycle stability of 211.3 mAh g^−1^ after 2000 cycles at 0.5 A g^−1^ [128]. Weng et al. reported the synthesis of a C/g-C_3_N_4_ composite via a simple one-pot synthesis approach, and the composite delivered a capacity of 254 mAh g^−1^ at 0.1 A g^−1^ and 160 mAh g^−1^ at 0.4 A g^−1^ (see Fig. 20g) [53]. Recently, Chen et al. showed that coating the surface of copper metal current collector with a thin film of 2D-C_3_N_4_ can improve Na storage by inhibiting unwanted surface interaction with the liquid electrolyte. The Na^+^ storage mechanism is illustrated by the scheme in Fig. 20h. At an areal current density of 0.013 mA cm^−2^, a high areal capacity of 0.036 mAh cm^−2^ was achieved [129].

**Fig. 21:**
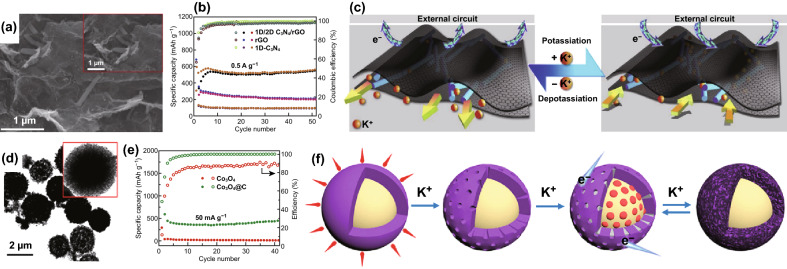
**a** SEM image of 1D/2D C_3_N_4_/rGO. **b** Cycling performances and coulombic efficiency of 1D-C_3_N_4_, rGO and 1D/2D C_3_N_4_/rGO at of 0.5 A g^−1^. **c** Possible mechanism of potassium storage in the 1D/2D C_3_N_4_/rGO composite. Reproduced with permission from Ref. [54]. **d** TEM and **e** comparison of the cycle life of Co_3_O_4_ and Co_3_O_4_@N–C at 50 mA g^−1^. f Scheme illustrating suggested potassiation and depotassiation mechanism of the Co_3_O_4_@N-C electrode. Reproduced with permission from Ref. [108]. Copyright permissions from Elsevier and American Chemical Society. (Color figure online)

In the case of potassium-ion batteries (PIBs), we showed that the high K^+^ affinity of 1D-C_3_N_4_ fiber facilitates high initial PIBs capacity, but it suffers from poor cycle life and DFT study showed that this was due to the high potassium diffusion barrier and poor conductivity. Guided by this theoretical discovery, we combined 1D-C_3_N_4_ fiber with rGO and fabricated a 1D/2D C_3_N_4_/rGO composite (Fig. 21a—SEM) which exhibited a larger surface area, superior K^+^ diffusivity, and improved conductivity. The composite delivered a superior reversible capacity 557.4 mAh g^−1^ after 50 cycles with impressive cycle stability (Fig. 21b). The K-ion storage mechanism is depicted in Fig. 21c and shows the bi-directional transport of ions through the interlayer of the composite [54]. In our recent work, we resolved the inadequate interlayer spacing of Co_3_O_4_ by coating it with N-doped carbon sourced from 1D-C_3_N_4_ fiber. The Co_3_O_4_@N–C possessed a core–shell morphology (Fig. 21d) in which N–C at the surface of Co_3_O_4_ ensured effective transfer of attracted K^+^ to the Co_3_O_4_ core. The composite delivered a superior reversible capacity of 448.7 mAh g^−1^ unlike pure Co_3_O_4_ spheres which only recorded ~ 10 mAh g^−1^ after 40 cycles (Fig. 21e). The potassium storage mechanism of the composite is depicted in Fig. 21f.

### Lithium-Sulfur Batteries

**Fig. 22:**
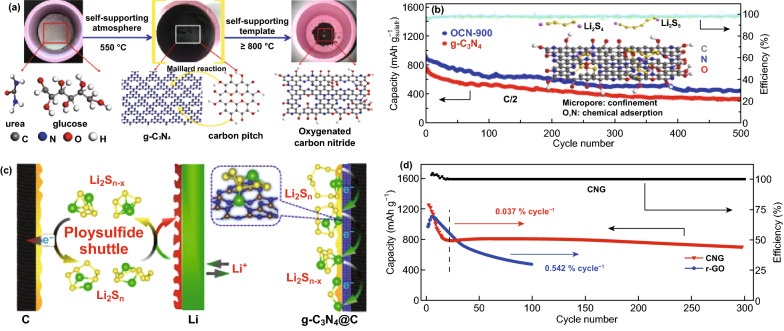
**a** Schematic illustration of graphene-like oxygenated carbon nitride (OCN) prepared by one-step self-supporting solid-state pyrolysis. **b** Cycling performances of the OCN sample prepared at different conditions and g-C_3_N_4_ at C/2 after initial activation process to allow complete access of the electrolyte to the active material. Inset: OCN with adsorbed LiPSs molecules. Reproduced with permission from Ref. [136]. **c** Schematic of the mechanism for polysulfide adsorption by the g-C_3_N_4_@CFM cathode. Reproduced with permission from Ref. [67]. **d** Cycling performance of LiPSs on CNG and r-GO. Reproduced with permission from Ref. [57]. Copyright permissions from American Chemical Society and Wiley–VCH. (Color figure online)

Carbon nitrides have proven to be effective at anchoring LiPSs in Li–S batteries, thereby inhibiting their dissolution in the electrolyte and boosting overall battery performance [134, 135]. Experimental battery studies on CNBMs as sulfur hosts for Li–S battery have confirmed the DFT predictions discussed earlier. For example, Liu et al. reported the application of a 2D graphene-like oxygenated C_3_N_4_ via a scalable one-step self-supporting solid-state pyrolysis process (Fig. 22a). The oxygen-rich functional groups possess strong chemical adsorption toward sulfur atoms; hence, they can inhibit the dissolution of polysulfides in Li–S electrolyte. As a cathode material, the resultant electrode delivered an initial columbic efficiency of 98% (S utilization of 84%) with a reversible capacity of 1407.6 mAh g^−1^ at C/20 (Fig. 22b) thanks to the surface functional groups [136].

Carbon coating for anchoring soluble polysulfides is an effective strategy to improve the conductivity of electrode materials in Li–S batteries [135]. Based on the conclusion of DFT calculations, Liao and co-workers showed that coating g-C_3_N_4_ on carbon-fiber mesh can serve as an effective anchoring strategy for LiPSs, and the g-C_3_N_4_@CFM electrode delivered 905 mAh g^−1^ at 0.1 C. Figure 22c shows the polysulfide adsorption mechanism of the composite electrode [67]. Liang et al. deployed polymeric C_3_N_4_ (p-C_3_N_4_) as a suitable cathode material to effectively attract LiPSs in common electrolyte solvents (1,3-dioxolane (DOL) and 1,2-dimethoxyethane (DME)). DFT calculations proved that interaction between p-C_3_N_4_ and LiPSs can alter the bonding and spatial configuration of the lithium polysulfides, which in turn tunes their redox kinetics. The results of Li–S battery testing in Fig. 22d showed that the electrode consisting of p-C_3_N_4_ and rGO (CNG) performed better than its individual constituents, improved stabilization, and kinetics of LiPSs and restrained shuttling effect [57].

**Fig. 23:**
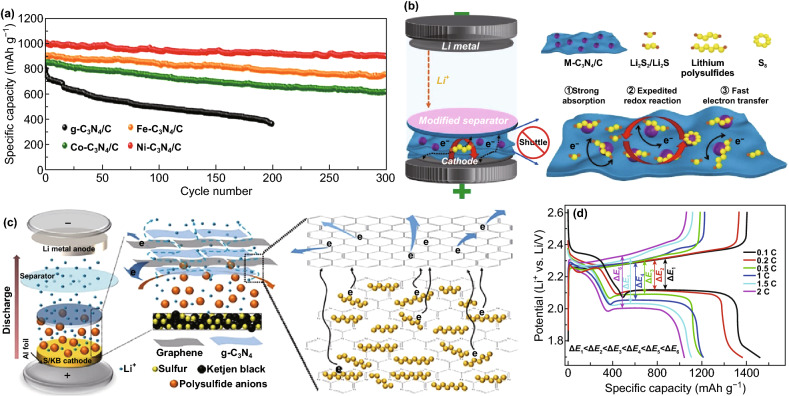
**a** Cycling performance of the heteroatom-doped-C_3_N_4_/C-modified separators at 0.5 A g^−1^. **b** Schematic illustration of the M-C_3_N_4_/C-modified separator to suppress the shuttle of polysulfides and expedite conversion reaction kinetics of polysulfides. Reproduced with permission from Ref. [69]. **c** Schematic of cell configuration with a laminated structure g-C_3_N_4_/GS cathode interlayer. **d** Charge/discharge profiles for the S/KB@C_3_N_4_/GS cathode at various scan rates. Reproduced with permission from Ref. [137]. Copyright permissions from Elsevier and Wiley–VCH. (Color figure online)

Coating of the commercial polypropylene separator with g-C_3_N_4_ is another strategy to facilitate LiPSs adsorption and effectively suppress polysulfide dissolution [134, 135]. Fan et al. reported the blending of commercial separator with carbon black (g-C-coated) and coating it on commercial glass fiber separator for LIPSs adsorption. The g-C_3_N_4_-coated separator showed better capacity retention in that after 400 cycles at 0.2 C, it could still deliver 773.2 mAh g^−1^, while the carbon-coated separator only had 611 mAh g^−1^ [68]. Chen et al. showed that coating of a transition metal (Fe, Ni, Cu, and Co) coordinated g-C_3_N_4_/C commercial separator improved LiPSs adsorption and performance. Experimental Li–S battery testing and DFT calculations concluded that Ni-modified C_3_N_4_ facilitated the LiPSs adsorption the best; hence, it outperformed the other metal modified C_3_N_4_/C-coated separators in terms of battery performance (Fig. 23a). A schematic of the polysulfide adsorption mechanism of the metal modified electrode is depicted in Fig. 23b. In terms of CNBCs, Qu et al. reported the use of graphene as a conductive material combined with C_3_N_4_ to form a g-C_3_N_4_/GS interlayer for a sulfur filled kejten black cathode for Li–S battery. Due to the electrostatic forces present as a result of *π*–*π* bonding between both materials, graphene and g-C_3_N_4_ heterogeneously intercalated with each in a closely packed fashion to form a laminated channel which inhibited diffusion of LiPSs toward the anode (Fig. 23c). The cathode material delivered a high reversible capacity (> 1200 mAh g^−1^) when cycled at 1 C and retained up to 50% of its capacity after 100 cycles. Moreover, from Fig. 23d a stable and consistent charge/discharge profile was maintained even when the electrode was cycled at various current densities [137]. To summarize the discussion on Li–S, CNBMs have been used for several functions in Li–S batteries and they have shown great promise as advanced electrode materials (Table 4). However, the percentage of S loading needs to be increased from an average of 50% to an average of ~ 80% for all CNBCs reports. Also, the binding mechanism and chemical interactions between the CNBMs and the binder need to be further investigated.

**Table 4:** CNBMs (pure, doped, and CNBCs) for different functions in Li–S batteries

Battery function	Materials	Synthesis	Morphology	Initial capacity (mAh g^−1^)	Sulfur loading	Reversible capacity (mAh g^−1^)	References
Cathode	OCN	One-step self-supporting solid-state pyrolysis (OSSP)	Free-standing 2D sheets	1407.6 @ C/20	56 wt%	447.3 @ 500 cycles	[177]
	GCN	Chemical deposition method	Sheet-like morphology	1250.1 @ C/0.05	70.4 wt%	578.0 @ 750 cycles	[132]
	C-NC/GN/g-C_3_N_4_	Low-temperature dissolution and high-temperature carbonization	3D interconnected network ultrathin sheets	~ 1270 @ 0.5 C	~ 78.1 wt%	~ 1130 @ 500 cycles	[178]
	CN@NSHPC	Double-solvent approach with controllable pyrolysis	Solid spheroidal nanoparticle	1099 @ 0.1 C	73% (1.7–2 mg/cm^2^)	445 @ 500 cycles	[179]
	CN/MoS_2_	Hydrothermal method	Laminar nanosheet	1252 @ 0.5 C	4 mg/cm^2^	680 @ 200 cycles	[184]
	rGO/g-C_3_N_4_/CNT	Ethanol-assisted spray-drying method	Microsphere-like structure	1030 @ 0.2 C	1.5 mg/cm^2^	820 @ 200 cycles	[182]
	3D PCN@rGO	Hydrothermal and freeze-drying	Interconnected sheets stacked by several layers	1240 @ 0.2 C	~ 1.5 mg/cm^2^	1005 @ 170 cycles	[181]
Sulfur host	C_3_N_4_@CFM	In situ coating method	Carbon fiber mesh	905 @ 0.1 C	0.6 mg/cm^2^	801 @ 100 cycles	[140]
	C_3_N_4_/rGO aerogel (CG12)	Hydrothermal, freeze-drying method	Inter-connected thin nanosheets	1024 @ 0.5 C	80 wt% (~ 1 mg/cm^2^)	770 @ 100 cycles	[138]
	MoS_2_/g-C_3_N_4_	Melt-diffusion method	Multi-layered sheet like structure	943 @ 1 C	~ 1.5 mg/cm^2^ (59.1%)	569 @ 400 cycles	[102]
Facilitate LiPS redox	CNG (Polymeric g-C_3_N_4_ and rGO)	Simple cyro-drying and annealing process	Interconnected thin sheets	~1250 @ ~ 330 mA/g	~ 0.6 mg of sulfur on 1.2 mg of CNG or r-GO	over 700 @ 280 cycles	[131]

Separator coating for Li–S battery							
Battery type	Materials	Synthesis	Morphology	Initial capacity (mAh g^−1^)	Sulfur loading	Reversible capacity (mAh g^−1^)	References
LiPS-trapping separator	C_3_N_4_/CNT@PP	Thermal condensation and oxidization etching with simple filtration process	Long-range continuous framework	~1350 @ 0.1 C	7.2 mg/cm^2^	870.5 @ 100 cycles	[180]
	g-C_3_N_4_	Thermal condensation and oxidization etching	Ultrathin nanosheet	~1000	− 4 mg/cm^2^	829 @ 200 cycles	[183]
	g-C_3_N_4_-C	Polymerization of melamine	Uniform and porous sheet layer	~1500 @ 0.2 C	1.7 mg/cm^2^	773.2 @ 400 cycles	[141]
	Ni-C_3_N_4_/C	Template assisted thermal polymerization	Sheet-like morphology	999 @ 0.5 A/g	0.5 mg/cm^2^	893 @ 300 cycles	[142]
Interlayer for LIPs	2D g-C_3_N_4_/Graphene	Ultrasonic treatment	2D micro sized shaped flakes	~1600 @ 0.2 C	50.9 wt%	710.4 @ 100 cycles	[137]
	Fe_3_O_4_/C_3_N_4_	Ultrasonication	Nanospheres coated tubular structure	1255 @ 0.2 C	1.0–6.4 mg/cm^2^	1048 @ 100 cycles	[180]

### Lithium-Oxygen Batteries

As discussed earlier, DFT predictions show that carbon nitrides are promising for Li-O_2_ battery because they possess a superior overpotential than most commercial electrodes because molecular level interaction with LiO_2_ indicates that they form a Li-N bonding which is dependent on the ratio of N in the material and greatly influences their overpotential [60].

Experimental study of pure and CNBCs in Li-O_2_ batteries has been reported in the literature. For instance, Liu et al. showed that g-C_3_N_4_ due to the lower conduction band (CB) potential of g-C_3_N_4_ and its low bandgap of ~ 2.7 eV the use of g-C_3_N_4_ together with an I^−^ ion redox mediator and non-aqueous electrolyte resulted in a drastic decrease in charging voltage [139].

**Fig. 24:**
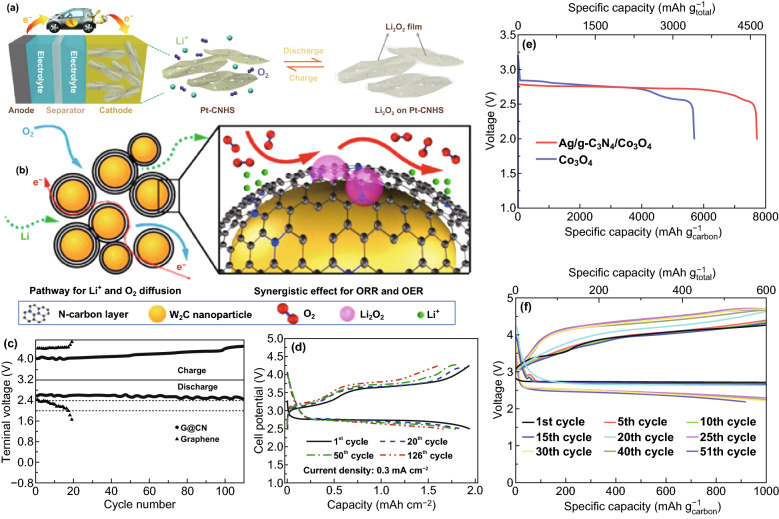
**a** Schematic illustration for the reaction process during cycling. Reproduced with permission from Ref. [63]. **b** Schematic diagram of the synergistic effect of N-doped carbon layer and W_2_C nanoparticles as Li-O_2_ battery catalyst. Reproduced with permission from Ref. [142]. **c** Comparison of the cycling performances of graphene and G@CN free-standing macroporous electrode. Reproduced with permission from Ref. [143]. **d** Cycle performance of RuO_2_@m-BCN in Li-O_2_ batteries with a current density of 0.3 mA cm^−2^. Reproduced with permission from Ref. [144]. **e** First discharge curves of Co_3_O_4_ and Ag/g-C_3_N_4_/Co_3_O_4_ as catalysts at a current density of 100 mA g^−1^ f First charge/discharge curves of the Ag/g-C_3_N_4_/Co_3_O_4_ as a catalyst at a current density of 500 mA g^−1^. Reproduced with permission from Ref. [145]. Copyright permissions from Springer Nature, Elsevier, Wiley–VCH, and Royal Society of Chemistry. (Color figure online)

Heteroatom doping (especially single atom doping) is a well-known method of modulating the electronic properties [140, 141]. Zhao et al. reported the application of a Pt-doped g-C_3_N_4_ (CNHS) for Li-O_2_ battery, and the performance and cycle stability of Pt-g-C_3_N_4_ (Pt-CNHS) exceeded those of pure holey g-C_3_N_4_. A schematic illustration of the Li-O_2_ reaction process is presented in Fig. 24a. Also, Li-O_2_ battery analysis (charge/discharge voltage plateau) shows that Pt-CNHS possess a lower overpotential when compared to CNHS with a round trip efficiency of 69% compared to 65% for CNHS [63]. Gao et al. also reported the design of a tungsten carbide-modified N-doped defective carbon (W_2_C@NC), and the composite exhibited a significant synergistic interaction (Fig. 24b) and delivered an initial reversible capacity of 10,976 mAh g^−1^ at a current density of 100 mAh g^−1^ with a low overpotential and long cycle life [142].

Luo et al. reported the fabrication of a 3D free-standing graphene@g-C_3_N_4_ (G@CN) composite which delivered superior performance (~ 17,300 mAh g^−1^ at a discharge voltage plateau of 2.6 V), energy efficiency (71%), and stable cycle life compared to the graphene electrode when applied in Li-O_2_ battery (Fig. 24c) [143]. Lou et al. also reported the application of mesoporous boron-doped carbon nitride (*m*-BCN) as a support for even distribution of ruthenium oxide (RuO_2_ nanoparticles). As a Li-O_2_ cathode, the RuO_2_@*m*-BCN composite displayed a superior discharge capacity (2.57 mAh cm^−2^ = 512 mAh g^−1^ based on the mass of the composite), smaller overpotential (0.18—discharge and 0.54—recharge), and excellent cycle stability even after 126 cycles. The charge/discharge curve for the composite when cycled at 0.3 mA cm^−2^ is presented in Fig. 24d [144]. Guo and co-workers reported the synthesis of a ternary composite of Co_3_O_4_-modified Ag/g-C_3_N_4_ for Li-O_2_. The synergistic interaction between these three constituents produced a composite with superior battery performance and catalytic activity with stable cycle life, high reversible capacity, and round-trip efficiency (Fig. 24e, f) [145].

### Lithium-Metal Batteries

**Fig. 25:**
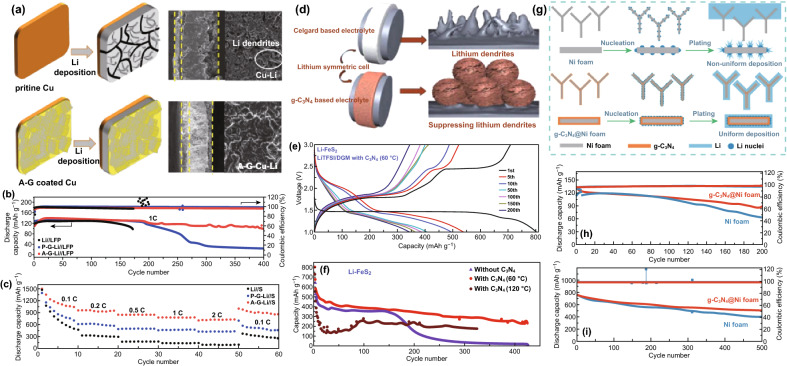
**a** Schematic illustration and SEM images of the cross-sectional view of Li deposition on pristine Cu and A-G-Cu electrodes before cycling and after depositing 5 mAh cm^−2^ of Li. **b** Cycle performance of the Li//LFP full cells with Li, P-G-Li, and A-G-Li. **c** Rate capability and of the Li//S full cells with Li, P-G-Li, and A-G-Li. Reproduced with permission from Ref. [151]. **d** Scheme of Li dendrite growth and inhibition depending on Li symmetric cells with g-C_3_N_4_ or without addition. **e** Galvanostatic charge/discharge curves of Li/FeS_2_ cell based on LiTFSI-DGM-C_3_N_4_ electrolyte of at 0.1C in a voltage range of 1—3 V. **f** Cycling performance of LiTFSI-DGM-C_3_N_4_-based Li/FeS_2_ cell (red circles) and its comparison with Li/LiTFSI-DGM/FeS_2_ cell. Reproduced with permission from Ref. [152]. **g** Schematic of the Li nucleation and plating process on Ni foam and g-C_3_N_4_@Ni foam. **h** Discharge capacity and CE of Li@g-C_3_N_4_@Ni foam|LiCoO_2_ and Li@Ni foam|LiCoO_2_ cells at 1.0 C. i Discharge capacity and CE of Li@g-C_3_N_4_@Ni foam|S and Li@Ni foam|S cells at 1.0 C. Reproduced with permission from Ref. [153]. Copyright permissions from American Chemical Society and Wiley–VCH. (Color figure online)

Despite the benefits of lithium metal as a preferred anode for high energy batteries, its large-scale application is hampered by two major issues: performance and safety [1, 146, 147]. These two problems are related in that they are both caused by Li dendrite growth. Moreover, other issues such as formation of SEI film [148–150] and non-uniform Li^+^ deposition are still of concern. These issues have been tackled in different ways, and because of the surface charges and functional groups of g-C_3_N_4_, it has been considered a very useful material for these various solutions. For instance, Luan and co-workers applied O- and N-rich graphene-like g-C_3_N_4_ as an effective artificial protective layer for Li-metal anode in half and full LIB and Li–S cells. Pristine g-C_3_N_4_ (P-G) and acid-treated g-C_3_N_4_ (A-G) were tested for Li-metal, and the A-G-Li exhibited the best wettability, structural stability, and least contact angle of almost zero indicating uniform Li^+^ distribution (Fig. 25a). From Fig. 25b, c, A-G-Li delivered the highest reversible capacity and superior capacity retention (80%) in both battery tests (LIB and Li–S, respectively) [151].

Considering the lithophilic nature of g-C_3_N_4_, its incredible mechanical strength, and unique morphology, Hu et al. reported the use of porous g-C_3_N_4_ microspheres as a polymeric electrolyte filler for lithium metal anode. Li dendrite was successfully suppressed in the g-C_3_N_4_ filler (Fig. 25d). It delivered superior performance and stability to the commercial electrolyte (Fig. 25e, f) [152]. Luo et al. reported the uniform coating of g-C_3_N_4_ on a 3D Ni foam (Fig. 25g) to design a g-C_3_N_4_@Ni foam which was applied as a current collector for Li-metal anode. The g-C_3_N_4_@Ni foam anode delivered superior capacity (Fig. 25h, i) to the pure Ni foam with excellent coulombic efficiency (98%), cycle stability, and capacity retention (72.9% at 1 C after 200 cycles) [153].

### Zinc-Air Batteries

**Fig. 26:**
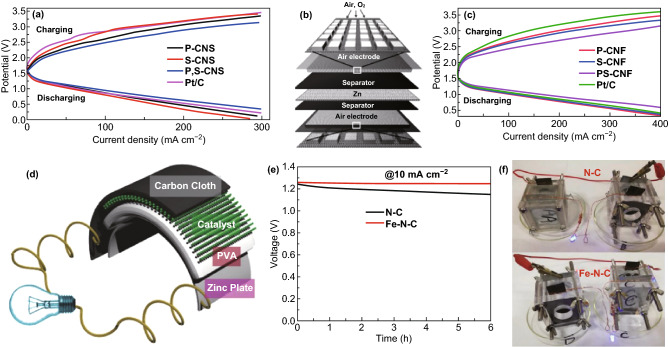
**a** Three-electrode ZABs charge and discharge polarization plots of commercial Pt/C, P-CNS, S-CNS, and P, S-CNS catalyst as air electrodes. **b** Schematic representation for the tri-electrode ZABs. Reproduced with permission from Ref. [158]. **c** Charge and discharge polarization curves for three-electrode ZABs with P-CNF, S-CNF, PS-CNF, and Pt/C catalysts as both air electrodes. **d** Schematic representation of the all-solid-state rechargeable Zn–air battery performance with N-GCNT/FeCo-3 acting as the air cathode. Reproduced with permission from Ref. [160]. **e** Galvanostatic discharge curves of the N–C or Fe–N–C cathode at the specific discharge current density of 10 mA cm^−2^. **f** Photograph of blue-light LED powered by two primary Zn-air batteries connected in series with N–C or Fe–N–C as the cathode catalyst. Reproduced with permission from Ref. [161]. Copyright permissions from Wiley–VCH, American Chemical Society, Elsevier, and Royal Society of Chemistry. (Color figure online)

The affordability, safety, and high specific energy density of ZABs are some of the reasons for the intensive research into this type of metal-air battery technology. However, the high cost and stability of metal-based catalysts for ZABs application [154, 155] have motivated researchers into designing metal-doped carbon materials [156, 157]. The high nitrogen content of C_3_N_4_ makes it a viable material for composite design or as a reliable precursor for N-doped carbon synthesis. For instance, Shinde et al. developed a 3D carbon NP sandwiched in phosphorus and sulfur co-doped g-C_3_N_4_ as a metal-free hybrid cathode material for ZAB. Electrochemical battery testing shows that the hybrid air–cathode operated at a voltage of approximately 1.51 V (Fig. 26a), delivered a high reversible capacity of 830 mAh g^−1^ along with a high energy density of 970 Wh kg^−1^, 198 mW cm^−2^ power density with excellent stability even after been recharged more than 210 h [158]. The composite was also tested in a tri-electrode ZABs system, and a schematic of the tri-electrode ZABs is shown in Fig. 26b.

Shinde et al. also synthesized 3D phosphorus, sulfur co-doped carbon nitride nanofibers. As a cathode material in a primary zinc-air battery, the hybrid nanofiber architecture operated at a 1.49 circuit voltage (consider Fig. 26c) and delivered a reversible capacity of 698 mAh g^−1^, power and energy density of 231 mW/cm^2^ and 785 Wh/kg, respectively, with great stability even after 240 h of operation [159, 160]. Ma et al. designed a flexible ZAB with bimetallic iron and cobalt (FeCo) sandwiched in N-doped carbon nanotubes (N-CNT). As an air–cathode in ZAB (Fig. 26d), the FeCo/N-CNT composite operated at an open-circuit voltage of 1.25 V, delivered a power density of 97.8 mW cm^−2^, and showed excellent stability at 100 mA cm^−2^ even after 144 cycles [160]. Zhang et al. reported the synthesis of a zinc air electrode using g-C_3_N_4_ as a template for N-doped carbon. The Fe–N-C air cathode operated at an open-circuit potential of 1.51 V, delivered a power density of 100 mW cm^−2^ and was able to power a 3 V blue LED lamp. As seen in Fig. 26e, the air–cathode operated with excellent stability at a current density of 10 mA cm^−2^ even after 6 h outshining the N–C electrode. The cathode materials were able to light up LED lamps; photographs of the LED lights powered by the cathode materials are presented in Fig. 26f [161].

### Solid-State Batteries

**Fig. 27:**
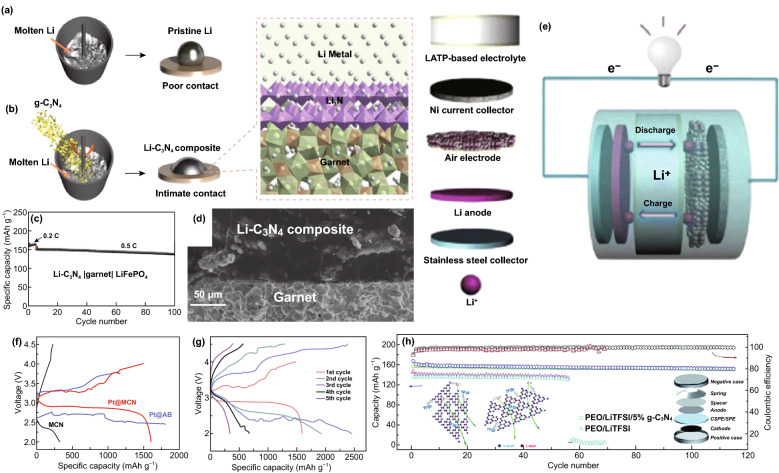
**a** Schematic illustration of the synthesis process of Li-C_3_N_4_ composite and the interfacial contact comparison of Li/garnet and Li-C_3_N_4_/garnet. **a** Garnet SSE presents a lithiophobic surface that forms pristine Li droplets. **b** Li-C_3_N_4_ composite shows an intimate contact with garnet and a Li_3_N layer is in situ formed at the interface. **c** Cycling performance of Li-C_3_N_4_|garnet|LiFePO_4_ full cell at a current rate of 0.5 C. **d** SEM images of the cross sections of Li-C_3_N_4_/garnet interfaces. Reproduced with permission from Ref. [165]. **e** Scheme of the configuration of the MCN-based electrode solid-state lithium-air battery. **f** Charge and discharge curves of the first cycle of the prepared air electrodes at a current density of 0.02 mA cm^−2^. **g** Cycling performance of Pt@MCN electrode at a current density of 0.02 mA cm^−2^. Reproduced with permission from Ref. [166]. **h** Capacity and coulombic efficiency versus cycle number for solid-state batteries using SPE and 5% g-C_3_N_4_ CSPE at 0.2 C rate. Inset: Schematics of enhanced lithium-ion migration in g-C_3_N_4_ CSPE. Reproduced with permission from Ref. [167]. Copyright permissions from Wiley–VCH, Springer Nature and Royal Society of Chemistry. (Color figure online)

Unlike liquid organic electrolyte systems, the possibility of maximizing electron transfer in a solid-state electrolyte and lithium metal battery system is rather arduous because of the reducing property of Li metal when it interacts with most solid-state electrolytes [162]. This reductive process occurs because of the high reduction potential of solid-state electrolytes against that of Li, for example Li_10_GeP_2_S_12_ (1.71 V), Li_1.3_Ti_1.7_Al_0.3_(PO_4_)_3_ (2.17 V), and Li_0.33_La_0.56_TiO_3_ (1.75) [163, 164]. While the reductive potential of Li_7_La_3_Zr_5_O_12_ (0.05 V) is very far from that of Li, it possesses a lithiophobic surface which means that it will experience poor contact and great surface resistance with lithium metal anode. However, this problem can be solved by meticulously coating its surface with a lithophilic material, thereby making changing its lithiophobicity and making Li_7_La_3_Zr_5_O_12_ lithophilic. Huang et al. used this approach when they reported the in situ formation of a Li_3_N layer between Li and Li_7_La_3_Zr_5_O_12_ such that Li^+^ can be effectively conducted and Li dendrite formation suppressed. This Li^+^ conducting and electron-insulating Li_3_N layer was created because of the reaction between molten Li and g-C_3_N_4_ to form a Li-C_3_N_4_ composite such that the role of C_3_N_4_ is for interface formation between Li and Li_7_La_3_Zr_5_O_12_ (Fig. 27a, b). The composite delivered a high capacity and stable cycle performance when tested in a solid-state battery (Fig. 27c) and maintained a strong/intimate contact with the Garnet (Fig. 27d) [165]. Lu et al. also reported the application of mesoporous carbon nitride (MCN) and acetylene black (AB) as a support for in situ growth of Pt nanoparticles for an all-solid-state lithium air battery system. A schematic of the assembled MCN-based electrode solid-state lithium-air battery is presented in Fig. 27e. As a cathode material in all-solid-state Li-air battery, the Pt@MCN electrode synthesized by Lu et al. recorded a significant high round trip efficiency (87%) at a high discharge voltage of ~ 2.87 V along with a low charge voltage of ~ 3.3 V (Fig. 27f, g) [166]. Sun et al. reported the application of g-C_3_N_4_ as a polymeric filler to improve the coupling effect between the amorphous specie and coordination unit of a solid polymer electrolyte in solid-state batteries. When applied as an electrolyte with LiFePO_4_ in an all-solid-state battery, 60 °C an initial reversible capacity of 161 mAh g^−1^ was delivered, and after 100 cycles, 155 mAh g^−1^ was still retained (Fig. 27h) [167].

## Conclusions and Future Perspectives

A comprehensive overview of structure-guided synthesis/fabrication and DFT-guided application of CNBMs for energy storage devices has been discussed in this review. CNBMs, including pure, doped, and CNBCs, exhibit high mechanical strength, excellent structural stability, abundant nitrogen-rich active sites, and surface functionalities, which are important for energy storage devices. Through the DFT-guided approach, CNBMs with superior structural and electronic properties are designed and their electrochemical properties can be modulated for improved performance. Moreover, the structure-guided approach facilitates the synthesis and fabrication of pure/doped C_x_N_y_ materials and CNBC’s, respectively. The massive attention into CNBMs for electrochemical energy storage and conversion can be attributed to the combination of these two approaches, and they are paramount to the advancement in this field [140, 168–172].

Despite the significant progress made so far in the study of CNBMs for energy storage devices, there are still unresolved issues; therefore, we provide some perspectives to resolve them. Firstly, the important preconditions must be considered when carrying out a DFT-guided synthesis/design of CNBMs. These prerequisites include the crystal phase and lattice parameter of the material, the interlayer distance (if it is a multi-layered material) and the exposed surface facet if it is a single-layered material, the ratio of C/N or other elements and type of nitrogen (pyridinic or graphitic-N) dominant in the structure of the material. These preconditions are critical when designing a material via a DFT-guided approach. Secondly, a clear and concise investigation of the metal ion storage mechanism of CNBMs is essential. This will provide a fundamental/atomic-scale understanding of the electrochemical processes occurring at the surface and carbon matrix of the CNBMs. It will also show which surface functionalities of CNBMs impact their metal ion storage mechanism and how the interfacial interactions can be optimized. Thirdly, in situ characterization of CNBMs is required for a deeper understanding of structural changes, surface functional group transformation, and metal ion storage. For example, through in situ Fourier-transform infrared spectroscopy (FTIR), functional groups such as hydroxyl, amine, and carboxylic groups on the surface of CNBMs can be observed. Phase and oxidation state changes can also be identified through in situ X-ray photoelectron spectroscopy (XPS) analysis. These advanced characterization techniques can provide crucial information that will enlighten researchers on the relationship between the electronic properties and the CNBMs. Lastly, theoretical/computational studies of CNBMs have proven to be fundamental in understanding their electronic properties for energy storage devices; hence, it must remain a crucial part of its future research. However, DFT is limited in studying CNBMs for energy storage device because it involves the use of metal atoms instead of ions, and it cannot effectively account for the effect of electrolyte, SEI layer formation, pulverization of electrode, the surface charge of materials and interfacial interactions. Therefore, improvement of existing DFT tools/functionals or the development of new ones that can account for these critical parameters is essential to achieve a closer alignment between DFT studies and experimental testing.

On a general note, the study of CNBMs is still at the early stage. Therefore, there is much room for improvement, and resolving these issues will surely propel the research to advance at a rapid pace.
